# Amodiaquine drug pressure selects nonsynonymous mutations in pantothenate kinase 1, diacylglycerol kinase, and phosphatidylinositol-4 kinase in *Plasmodium berghei *ANKA

**DOI:** 10.12688/openresafrica.13436.3

**Published:** 2023-10-19

**Authors:** Jean Chepngetich, Brenda Muriithi, Beatrice Gachie, Kevin Thiong'o, Mercy Jepkorir, Jeremiah Gathirwa, Francis Kimani, Peter Mwitari, Daniel Kiboi

**Affiliations:** 1 Department of Molecular Biology and Biotechnology, Pan African University Institute for Basic Sciences, Technology and Innovation, Nairobi, 62000, 00200, Kenya; 2 Centre for Traditional Medicine and Drug Research, Kenya Medical Research Institute, Nairobi, 54840, 00200, Kenya; 3 Centre for Biotechnology Research and Development, Kenya Medical Research Institute, Nairobi, 54840, 00200, Kenya; 4 Department of Biochemistry, Jomo Kenyatta University of Agriculture and Technology, Nairobi, 62000, 00200, Kenya

**Keywords:** Lumefantrine, Piperaquine, Amodiaquine, Resistance, Plasmodium berghei, kinase

## Abstract

**Background:**

Lumefantrine (LM), piperaquine (PQ), and amodiaquine (AQ), the long-acting components of the artemisinin-based combination therapies (ACTs), are a cornerstone of malaria treatment in Africa. Studies have shown that PQ, AQ, and LM resistance may arise independently of predicted modes of action. Protein kinases have emerged as mediators of drug action and efficacy in malaria parasites; however, the link between top druggable *Plasmodium* kinases with LM, PQ, and AQ resistance remains unclear. Using LM, PQ, or AQ-resistant *Plasmodium berghei* parasites, we have evaluated the association of choline kinase (CK), pantothenate kinase 1 (PANK1), diacylglycerol kinase (DAGK), and phosphatidylinositol-4 kinase (PI4Kβ), and calcium-dependent protein kinase 1 (CDPK1) with LM, PQ, and AQ resistance in *Plasmodium berghei* ANKA.

**Methods:**

We used *in silico* bioinformatics tools to identify ligand-binding motifs, active sites, and sequence conservation across the different parasites. We then used PCR and sequencing analysis to probe for single nucleotide polymorphisms (SNPs) within the predicted functional motifs in the CK, PANK1, DAGK, PI4Kβ, and CDPK1. Using qPCR analysis, we measured the mRNA amount of PANK1, DAGK, and PI4Kβ at trophozoites and schizonts stages.

**Results:**

We reveal sequence conservation and unique ligand-binding motifs in the CK, PANK1, DAGK, PI4Kβ, and CDPK1 across malaria species. DAGK, PANK1, and PI4Kβ possessed nonsynonymous mutations; surprisingly, the mutations only occurred in the AQr parasites. PANK1 acquired Asn394His, while DAGK contained K270R and K292R mutations. PI4Kβ had Asp366Asn, Ser1367Arg, Tyr1394Asn and Asp1423Asn. We show downregulation of PANK1, DAGK, and PI4Kβ in the trophozoites but upregulation at the schizonts stages in the AQr parasites.

**Conclusions:**

The selective acquisition of the mutations and the differential gene expression in AQ-resistant parasites may signify proteins under AQ pressure. The role of the mutations in the resistant parasites and their impact on drug responses require investigations using reverse genetics techniques in malaria parasites.

## Introduction

Malaria is a leading cause of death across many tropical and subtropical countries ^1^. Of the six species that infect humans, *Plasmodium falciparum* is the most virulent and significant cause of the majority of deaths ^2^. In 2020, at least 241 million malaria cases and 627,000 deaths were reported globally, with 95% in Africa ^1^. The disease disproportionally affects children under five and accounts for 77% of malaria-associated deaths ^1^. Currently, the recommended treatment for uncomplicated *Plasmodium falciparum* malaria involves using artemisinin-based combination therapies (ACTs), comprising two components: a short-acting artemisinin derivative and a long-acting partner drug ^1^. In Africa, three main ACTs are advocated: lumefantrine (LM)-artemether (ATM), piperaquine (PQ)-dihydroartemisinin (DHA), or Artesunate (ASN)-amodiaquine (AQ) ^1^. The artemisinin derivative rapidly eliminates the bulk of the parasites in two hours, while the long-acting drug kills the residual parasites over weeks ^3^.

Over the last two decades, the use of ACTs against *Plasmodium falciparum* has significantly decreased malaria-related deaths worldwide. However, the emergence of resistance to frontline artemisinins and partner drugs in Southeast Asia and Africa ^4– 7^, has threatened these gains made by the ACTs treatments. The stable parasite transmissions in many African countries, coupled with the extensive use of the ACTs, have consistently submitted prime pressure for selecting drug-resistant parasites, primarily against the long-acting drugs LM, PQ, and AQ ^8^. This has spurred a new impetus to interrogate shared and unique resistance mechanisms against LM, PQ, and AQ. Like chloroquine, AQ and PQ are aminoquinoline drugs predicted to exert their action within the parasite digestive vacuole by inhibiting toxic heme polymerization ^9^. However, PQ and AQ are active against selected CQ-resistant parasites, suggesting that these three drugs may differ in some modes of action and resistance mechanisms. These studies also indicate that the mechanisms of AQ, PQ, and LM may involve other unexplored essential parasite proteins. LM is an aryalcohol drug, predicted to share mechanisms of action with 4-aminoquinoline ^9^. However, recent studies suggest reciprocal polymorphisms between 4-aminoquinoline drugs and LM ^10, 11^. These studies indicate that while LM, PQ, and AQ may share some resistance mechanisms, unique drug-specific mechanisms also need further investigation. Several genes, associated mutations, and resistance mechanisms against LM, PQ, and AQ have been identified and validated ^8^. The shared genes between the LM, PQ, and AQ are polymorphisms or amplification of the chloroquine resistance transporter (crt) and multi-drug resistance 1 gene (mdr1) ^12, 13^. Recent studies have also identified new markers potentially unique for LM, PQ, and AQ. For instance, amplification and SNPs in *Plasmepsin 2/3* in *Plasmodium falciparum* are associated with PQ resistance ^14, 15^. Also, decreased susceptibility to PQ and recrudescence following DHA/PQ treatment is associated with an E415G substitution in the exonuclease ^16^. On the other hand, a mutation in cysteine desulfurase is associated with reducing susceptibility to LM in field isolates ^17^. These studies reveal new and unprecedented genes that directly or indirectly mediate LM, PQ, and AQ efficacy.

Studies on the discovery of antimalarial drugs with transcending action to the different life cycle stages of the malaria parasites have validated protein kinases in mediating drug action and resistance ^18– 22^. In *Plasmodium* species, kinases control signal transduction pathways that regulate essential cellular processes such as growth, development, and reproduction ^23, 24^. While a link between kinases and AQ, LM, or PQ in *Plasmodium* remains unclear, the effect of aminoquinoline drugs; chloroquine (CQ), and AQ on the function of kinases in other eukaryotic and cancer cells is well documented ^25– 27^. For instance, CQ and AQ significantly enhance the activity of cyclin-dependent kinase inhibitors in rheumatoid arthritis ^26^. As an anti-inflammatory drug, CQ affects the phosphorylation of extracellular signal-regulated kinases and the mitogen-activating protein kinase ^28^. There are divergences in protein kinases between other eukaryotes and *Plasmodium* species ^19, 29^. However, the ability of aminoquinoline drugs CQ and AQ to inhibit phosphorylation suggests a link between protein kinases and the action of the aminoquinoline antimalarial drugs. In *Plasmodium*, protein kinase C involved in the growth, maturation, and differentiation of all the asexual blood stages can be inhibited by CQ, but only in the CQ-sensitive *Plasmodium falciparum* ^30^, suggesting a possible association between the kinase with CQ susceptibility in the CQ resistant *P. falciparum*. In a separate study, CQ could competitively inhibit protein tyrosine kinase activity by blocking the protein substrate binding site ^31^. The ability of CQ to inhibit key kinase proteins implies that closely related antimalarial drugs, such as AQ and PQ, may exert their action through similar mechanisms.

In *Plasmodium* species, the protein kinases are drug activators, potential targets for the drug metabolites, and mediators of drug resistance ^22, 32– 34^. For instance, artemisinin inhibits parasite growth by blocking the *Plasmodium falciparum* phosphatidylinositol-3-kinase (PfPI3K), while an increase in PfPI3K levels confers artemisinin resistance ^35^. Polymorphisms in PANK1, the first enzyme in the coenzyme A (CoA) biosynthesis pathway, are associated with resistance to newly developed pathothenamides drugs ^22, 36^. Other kinase based-inhibitors that have progressed to human clinical trials are phosphatidylinositol 4-kinase (PI4K), with inhibitor 37 (MMV048) ^23, 37^. New multi-stage target drugs such as Imidazopyrazines block the ATP-binding pocket of PI4K, altering the intracellular distribution of phosphatidylinositol 4-phosphate ^38^. Commonly, genes and mutations mediating resistance to one class may be associated with cross-resistance to drugs of unrelated chemical classes and modes of action.

Here, we used *in silico* computational analysis, PCR, sequencing, and qPCR analysis to interrogate the association of five selected kinases with LM, AQ, and PQ resistance in *Plasmodium berghei* ANKA. The stable drug-resistant parasites used in this study had previously been selected by submitting *P. berghei* ANKA parasites separately to LM, AQ, or PQ for at least 40 mechanical passages ^39– 41^. We reveal nonsynonymous mutations in Pantothenate kinase 1 (PANK1) (PBANKA_1022600), Diacylglycerol kinase (DAGK) (PBANKA_1334600), and Phosphatidylinositol 4-kinase beta (PI4Kβ) (PBANKA_1109400), only in the AQr parasites. The mutations either occurred in ligand binding motifs or within active sites of the enzymes and thus may impact the function of the kinases. We further report differential mRNA expression profiles of the PI4Kβ, PANK1, and DAGK across the LM, AQ, and PQ resistant parasites.

## Methods

### Ethical consideration

This study was conducted at KEMRI, Nairobi, Kenya. Permission to use laboratory animals was granted under the approvals SERU No KEMRI/CTMDR/SERU/P092/4096 on 20 ^th^ November 2020 and KEMRI ACUC/02.06.2021. To ameliorate any suffering of the laboratory animals used in the study, we adhered to the ARRIVE Guidelines. Experimental mice were monitored daily for any severe illness or injury to alleviate pain and suffering and avoid mortality associated with parasite infection. No animal became severely ill before the experimental endpoint (day nine post parasite infection for drug assays). At the end of each study, mice were euthanized using carbon dioxide (CO _2_) inhalation. CO _2_ exposure was delivered from compressed gas cylinders using a gradual fill method at a displacement rate of 20% of the chamber volume per minute. ARRIVE Guidelines checklist and the detailed report on the use and care of laboratory animals used in the study are deposited in OSF ^42^.

### *In silico* computational analysis by multiple sequence alignment and I-TASSER

PlasmoDB and UniProt were used to retrieve protein sequences for the CK, PANK1, DAGK, PI4Kβ, and CDPK1. The evaluated protein kinases were derived from six different parasite species; two species that infect humans; *Plasmodium falciparum* 3D7, *Plasmodium vivax* P01, two species that infect the primates: *Plasmodium adleri* G01, and *Plasmodium reichenowi* G01) and two species that infect rodents: *Plasmodium berghei* ANKA, *Plasmodium chabaudi chabaudi*). Multiple sequence alignments of the retrieved proteins were performed using CLUSTAL OMEGA. Mapping of conserved regions and prediction of structure, function, and ligand binding motifs were queried using the protein structure prediction software Alpha Fold ^42^, MOTIF search, Phyre2 version 2.0 ^43^, and ITASSER version 5.1 ^44, 45^.

### Parasites, host, and compounds

Two parasite reference lines of *P. berghei* ANKA (the MRA-865 and MRA-868 reference lines obtained from MR4, ATCC ^®^ Manassas, Virginia) were used as the drug-sensitive wild-type (WT) parasites. Also, stable multidrug-resistant parasites: the PQ-resistant (PQ ^R^) *P. berghei* ANKA parasites previously selected from the MRA-865 line, the LM-resistant (LM ^R^), and AQ-resistant (AQ ^R^) *P. berghei* ANKA parasites previously selected from MRA-868 line ^39, 41^, were utilized. Male *Swiss albino* mice weighing 20±2g outbred at KEMRI Animal house Nairobi were employed, housed, and fed as previously described ^46^. LM, PQ and AQ were donated from Universal Corporation, Kikuyu, Kenya. All drugs, PQ, LM, and AQ were freshly prepared and administered as previously described ^39^.

### Baseline drug profiles of the resistant parasites

The drug susceptibility profiles of the WT, LM ^R^, AQ ^R^, and PQ ^R^ parasites were assessed in the standard 4-day suppressive test (4-DT) ^47^. Infection, randomization of experimental mice, drug administration, estimation of parasite densities, and calculation of the percentage of drug killing was performed and determined as previously described ^46, 48^. At least three mice were used for each drug dosage and placebo-treated control. On day zero (D0), the mice were randomly infected with 1×10 ^6^ parasitized red blood cells (PRBCs); the mice were then orally treated with either 2.5, 5, 10, 20, 40, or 50mg/kg of drug concentrations at 4 hrs, 24hrs, 48hrs, and 72hrs post parasite inoculation. Each mouse's percentage of parasite density was estimated using a microscope ×100 magnification. Briefly, 10 different fields per smear on a slide from each mouse were counted with each field consisting of approximately 100 red blood cells, translating to coverage of 1000 total red blood cells per smear. Each drug dosage's percentage of drug killing ability was calculated as previously described ^47^.

### Extraction of DNA, PCR amplification, and sequencing

DNA was extracted from WT, LM ^R^, AQ ^R^, and PQ ^R^ parasites in at least 1 ml of the infected blood isolated from a mouse with a parasitemia of 5 – 10%. Blood was spun at room temperature for 2 minutes at 8,000xg to pellet the infected red blood cells. The supernatant was discarded, and the pellet was resuspended in a 30 ml volume of cold 1× Red blood cells lysis buffer (Thermo Fischer Scientific™ Cat No 00-4333-57) for 30 minutes, followed by spinning at 4,000 rpm for 10 min at room temperature. The parasite pellet was washed twice with 30 ml 1×PBS with centrifugation at 4,000 rpm for 5 min at 4°C. Parasite genomic DNA (gDNA) was extracted using a commercial GeneJET Whole Blood Genomic DNA Purification Mini Kit (Thermo Fischer Scientific ^TM^ Cat No K0781) following the instructions from the manufacturer. Target regions in selected kinases were PCR amplified using the primers listed in Table 1. Briefly, 2 µl of pgDNA, 0.2 pmol/µl forward, and reverse primers were used in 25 µl reactions and amplified using the Q5 High Fidelity Master Mix (NEB™ Cat No M0494S). PCR products were analyzed in 2% agarose gel, purified using the AddBio PCR Purification Kit (addbio™ Cat No 10078), and sequenced based on BigDye v3.1 using a 3730xl sequencer based on BigDye v3.1.

**Table 1.** A list of oligonucleotide sequences used for PCR amplification and sequencing of selected regions of the *Plasmodium berghei* choline kinase *(ck)*, *Plasmodium berghei* pantothenate kinase 1 *(Pbpank1)*, *Plasmodium berghei* phosphatidylinositol 4-kinase beta *(Pbpi4kβ)*, *Plasmodium berghei* diacylglycerol kinase *(Pbdagk)*, and *Plasmodium berghei* calcium-dependent protein kinase 1 *(Pbcdpk1)*.

Name of the gene	Primer sequence (5’– 3’)	Position	Tm
*Pbck*_Forward	ACT TTA CCA AGC CAT TGG GAT	664 - 684	51.05
*Pbck_R*everse	GAT AAC CTC GAA TAA TCG ACC A	1210 - 1189	53.11
*Pbpank1*_Forward	ACC AAT ACA AAT CAA ATC ACC ACT	879 - 902	51.13
*Pbpank1*_Reverse	ACC TCC GAT AAC TCC TAA AAA TC	1644 - 1622	51.3
*Pbpi4kb*_ Forward	ATT CGA AAA ATA TCA CCT TAT GGC	3841 - 3864	51.62
*Pbpi4kb*_Reverse	CAT ATT GAA CAC TTC GGA AGT TG	4624 - 4602	51.96
*Pbdagk*_Forward	GGT GAT GGA ACT TTA AAT TGG GTT	406 - 429	51.51
*Pbdagk* _Reverse	ATG CAA ATT CTA GCC CCA AAT C	1198 - 1177	52.23
*Pbcdpk1*_Forward	GCA GTT TGA TAA AGG AAG ATA TAG T	273 - 297	51.59
*Pbcdpk1*_ Reverse	AGC TTT TGG CTT CCT TCA AAT	1064 - 1044	51.78

### Sequence analysis and prediction of protein structure

DNA sequences were analyzed using CodonCode Aligner, Lasergene 11 Core Suite, and CLUSTAL Omega, available on the EBI website. Structure analysis of the mutated protein was done using Alpha Fold and compared with ITASSER for possible altered ligand binding sites, secondary structure comparison, and amino acid interactions.

### Schizont culture, purification, and synchronization of the parasites

Parasites were first synchronized for extraction of total RNA from the different asexual blood stages (ABS). Mice with ~5% ring-stage parasites were used as the donor for schizont culture *in vitro*. Briefly, 1–3 ml of aseptically collected infected blood was mixed with 72 ml of RPMI 1640 with 25 mM HEPES, 2mM L-Glutamine (Invitrogen™ Cat 52400-041), and 25ml of heat-inactivated Fetal Bovine Serum (Gibco™ Cat No 10500-64), 2 ml of NaHCO _2_ and 1 ml of Penicillin/Streptomycin (Gibco™ Cat No 15140-122). The parasite cultures were maintained at 37°C with gas mixture (5% O _2_, 5% CO _2,_ and 90% N _2_) for 22 hrs and 55 rpm. Thin smears were prepared from overnight schizont cultures before purification of the schizonts on a 55% Nycodenz (Progen Cat No 1002424)/PBS gradient. Mice were then infected intravenously each with approximately 10 parasites (10 schizonts) from the culture. Infected blood was collected after 18 hrs for late trophozoites and after 24 hrs for the schizonts stage. Parasite pellets were prepared by lysing the infected red blood cells in cold 1× Red blood cells lysis buffer (Thermo Fischer Scientific™ Cat No 00-4333-57) ^46^. Parasite pellets were immediately used to extract total RNA or resuspended in 100 µl of TRIzol™ reagent (Thermo Fischer Scientific™ Cat No 15596026) and frozen at -80 degrees.

### Extraction of RNA and synthesis of cDNA

Total RNA was prepared from approximately 1×10 ^6^ fresh parasites pellet. The pellets were first thawed and then resuspended by vortexing for 10 minutes at room temperature. This was followed by centrifugation for 1 minute at 4,000 rpm at 4°C to remove debris. The supernatant was transferred to a clean Eppendorf tube. To each sample, 200 µl of chloroform (Sigma-Aldrich™ Cat No C2432) was added, vortexed for up to 30 seconds, and left at room temperature for 10 minutes. Centrifugation followed for 10 minutes at 13,000x g at 4°C to separate the phases. The upper aqueous phase was transferred to a fresh tube, and 500 µl of isopropanol (Sigma-Aldrich™ Cat No SKU 19516) was added. The samples were allowed to precipitate at -20°C for 1 h. The RNA was pelleted by centrifugation at 4,000 rpm for 15 min at 4°C. The supernatant was decanted, the pellet washed with 1 ml of 70% ethanol, and then spun again for 10 minutes at 13,000x g at 4°C. The supernatant was decanted, and the pellet was dried on a hot plate for 3 – 5 min. The pellet was then resuspended in 90 µl RNase-free demineralized water. Total RNA was quantified by NanoDrop™ (Thermo Fischer Scientific™ Cat No ND 2000). The first-strand cDNA synthesis was performed in a final volume of 20 µl using Solis BioDyne FIREScript Reverse Transcriptase cDNA Synthesis KIT (Cat No: 06-15-00050) following instructions from the manufacturer. Briefly, 5 µg of total RNA, 1 µl of oligoDT (100 µM), and nuclease-free water were added with 2 µl of 10 x RT reaction buffer with DDT, 0.5 µl RiboGrip™ RNase inhibitor (40U/µl), 0.5 µl of Deoxyribonucleotide triphosphates (dNTPs) (20 mM each) and 1 µl of FIREScript ^®^ Reverse Transcriptase (10 U/µl) and mixed gently. The reaction mix was incubated at 65°C for 30 min, then at 85°C for 5 min to terminate the reaction, and finally chilled on ice. The synthesized cDNA was used as a template for qPCR assays.

### qPCR assays

Using the Pbβ-actin I as the reference gene, the relative expression of *pank1*, *dagk,* and *pi4k*β was performed in a final volume of 20 μl using HOT FIREPol ^®^ EvaGreen qPCR Mix Plus (ROX), 5x (Solis Biodyne Cat No: 08-24-0000S). Briefly, 4 µl of 5x HOT FIREPol ^®^ mix and 14 µl of water were mixed with 0.5 µl of forward and reverse primers each ( Table 2) and 1µl cDNA. The reaction mix was run for initial activation at 95°C for 12 min; denaturation at 95°C for 15 secs; annealing at 60°C for 20 secs for 40 cycles, and elongation at 72°C for 20 secs. The samples were run in triplicates using QuantStudio™ 5 System qPCR machine serial number: 272520210 (Thermo Fischer Scientific).

**Table 2.** A list of oligonucleotide sequences used in the qPCR assays to measure the mRNA expression levels of *Plasmodium berghei* choline kinase *(Pbck)*, *Plasmodium berghei* pantothenate kinase 1 *(Pbpank1)*, *Plasmodium berghei* phosphatidylinositol 4-kinase beta (Pbpi4kβ), *Plasmodium berghei* diacylglycerol kinase *(Pbdagk)*, and *Plasmodium berghei* calcium-dependent protein kinase 1 *(Pbcdpk1)* at the different asexual blood-stage (rings, early trophozoites, late trophozoites, and schizonts) in the resistant parasite relative to the wild-type drug-sensitive parasite with *Pbβ-actin I* as the reference gene using Maxima SYBR Green chemistry in qPCR.

Name of the gene	Primer sequence (5’– 3’)	Position	Tm
*Pbck*_ Forward	AAGT CAC AAA TAC CGT ATG ATC AA	1095 - 1119	51.57
*Pbck*_Reverse	GAT AAC CTC GAA TAA TCG ACC A	1189 - 1211	51.18
*Pbpank*1_ Forward	AAG GCG ATT TTC ATT TGA GAT C	1541 - 1562	51.23
*Pbpank*1_ Reverse	ACC TCC GAT AAC TCC TAA AAA TC	1622 - 1644	51.3
*Pbpi4k*β _ Forward	TTT CGA ATG GAA CAC AAT TTT GC	4490 - 4512	51.89
*Pbpi4k*β _ Reverse	CAT ATT GAA CAC TTC GGA AGT TG	4602 - 4624	51.96
*Pbdagk* _ Forward	GGT GAT GGA ACT TTA AAT TGG GTT	406 - 429	51.51
*Pbdagk* _ Reverse	ACC CAA ATG CAT TAG CAA AAT CA	504 - 526	52.59
*Pbcdpk1*_ Forward	TAA CAA AAG ATG TAC GGC TGA G	927 - 948	52.3
*Pbcdpk1*_ Reverse	AGC TTT TGG CTT CCT TCA AAT	1044 - 1064	51.78
*Pbβ-actin I* – Forward	CAGCAATGTATGTAGCAATTCAAGC	392 - 416	56.8
*Pbβ-actin* *I* - Reverse	CATGGGGTAATGCATATCCTTCATAA	523 - 498	58.9

### Statistical analysis

The percentage of parasitemia and drug killing in the different drug concentrations were analyzed using the nonparametric Mann-Whitney U test in the R statistical software version RStudio 2022.07.0 with a p-value set at 0.05. The means for the gene expression levels from three independent experiments and triplicate assays were compared using the Mann-Whitney U test with a p-value set at 0.05. The relative expression data were normalized using β-actin I as the reference gene based on the formula 2 ^ΔΔCT^ ^49^.

## Results and discussion

### *In silico* bioinformatics analysis reveals unique binding sites

Here, we have analyzed five kinases: choline kinase (CK), pantothenate kinase 1 (PANK1), diacylglycerol kinase, putative (DAGK), phosphatidylinositol 4-kinase beta (PI4Kβ), and calcium-dependent protein kinase 1 (CDPK1), and their association with AQ, LM and PQ resistance in *Plasmodium berghei* (Figure S1) ^42^. We identified the likely functional regions in the five kinases using multiple sequence analysis (MSA), ITASSER tools, and MOTIF finder. We prioritized these sites for targeted PCR amplification and sequencing.

Our MSA of the kinase protein reveals unique conservation of motifs across the different species suggesting a likely association with function. Conserved motifs are enriched enzyme’s active sites, ligand binding sites, functional groups, and plausible drug targets ^44, 45^. We further reasoned that the credible kinases that may associate with LM, PQ, and AQ resistance and drug responses are expressed within the ABS, the predicted sites of action of the drugs. The query on the expression profile of the five kinases from PlasmoDB shows a consistent expression across the different stages of the parasite's lifecycle, notwithstanding variation in the levels. To validate *in silico* bioinformatics analysis, we searched the previous link for each of the kinases as a drug target, drug resistance, or activator of the drug in malaria parasites. Several studies have validated the five kinases as drug targets in the malaria parasites ^34, 50– 52^. Mutations in regulatory and functional motifs and upregulation of kinases modify drug responses in malaria parasites ^22, 35, 53^. By querying the COACH and COFACTOR tools on the ITASSER platform using sequences from *Plasmodium berghei*, we identified the predicted functional, active sites, and ligand binding sites of the CK, PANK1, DAGK, PI4Kβ, and CDPK1 ( Table 3). ATP binding sites, present in all kinases, are highly druggable, and the structural requirements for designing potent ATP-competitive kinase inhibitors ^54^.

**Table 3.** A summary of the *in-silico* bioinformatics results from multiple alignments of the protein kinase sequences, MOTIF Search using genome.jp database ( https://www.genome.jp/tools/motif/), ligand binding, and active sites search using ITASSER online tools.

Name of the protein	Accession no	Number of motifs and regions of shared predicted by MOTIF Search	ITASSER ligand-binding sites/ active sites
choline kinase (CK)	PBANKA_1040100	3 motifs (136 – 348)	184 – 190, 284 – 305
pantothenate kinase 1 (PANK1)	PBANKA_1022600	2 motifs (66 – 153, 200 – 469)	130 – 188, 272 – 304
diacylglycerol kinase, putative (DAGK)	PBANKA_1334600	2 motifs, (120 – 179, 249 – 433)	154 – 186, 272 – 381
phosphatidylinositol 4-kinase beta (PI4KB)	PBANKA_1109400	3 motifs (350 – 475, 1240 – 1500)	1246 – 1305, 1359 – 1370
calcium-dependent protein kinase 1 (CDPK1)	PBANKA_0314200	14 motifs (50 – 350, 375 – 525)	145 – 263

To identify critical regions in the target kinases, we used reference protein sequences from *Plasmodium berghei* ANKA. Using the MOTIF finder, we found three predicted motifs in CK ( Table 3). As expected, the choline kinase motif was identified from amino acids 139 – 350 of the protein. The other motifs were APH and TCAD9, corresponding to amino acids positions 160 – 324 and 281 – 324, respectively. We then located binding motifs from amino acids 184 – 190 and 284 – 305 using the ITASSER tools. Here, we PCR amplified targeting positions 221 – 318 region comprising the protein's predicted binding and active sites. The target region of interest included four ligand-binding sites with the ADP-binding site and an essential ATP synthesis site at position 288 ( Table 3). As the first enzyme in the CDP-choline pathway or Kennedy pathway, CK catalyzes the initial step of the CDP- choline pathway and can regulate phosphatidylcholine, the most abundant phospholipid in *Plasmodium falciparum* ^55^. The binding of small molecules at the active site was shown to inhibit the function of CK, resulting in parasite killing ^52^. Therefore, it is likely that for parasites to escape killing by drugs and drug metabolites targeting the CK, mutations within the active site or its close proximity may modify binding affinities and offer protection from such drugs.

When we focused our analysis on PANK1, using the MOTIF finder, we located two fumble motifs ( Table 3). A functional fumble motif is critical for cell division and cell membrane synthesis ^56^ and thus an important region for mapping potential polymorphisms that may occur in resistant parasites. PANK1 is a proven drug activator ^57 ^and catalyzes the first step in coenzyme A biosynthesis, which involves the phosphorylation of pantothenate ^21^. Mutations in PANK1 alter its activity and responses to new antiplasmodial pantothenate analogs ^22^. Our analysis using ITASSER tools predicted four ligand-binding sites of importance in acetyl coenzyme A and adenosine 5-diphosphate. The region of interest in this study was within the predicted motifs (303 – 316) located in the fumble domain. We found consensus binding residues from 311 – 330 in all the top Protein Database (PDB) hits based on the COACH – ITASSER results, suggesting the essentiality of these residues in the functioning of the PANK1 protein. Acquisition of mutations within the fumble domain results in inactivation of the domain and significantly alters phospholipids biosynthesis and cell division ^56^, supporting our search for polymorphisms within this region.

Our *in silico* analysis of DAGK using the MOTIF finder predicted two motifs ( Table 3). As expected, we located the Diacylglycerol kinase accessory domain (249 – 433), and Diacylglycerol kinase catalytic domain (120 – 179). DAGK has catalytic and accessory domain motifs. The enzyme converts diacylglycerol to phosphatidic acid, which regulates exocytosis in apicomplexan, including *Plasmodium* species ^58^. Here, we mapped our target motif at positions 129 to 178 of the DAGK, which is within the highly conserved catalytic domain as predicted by the MOTIF search on the genome.jp database ( Table 3). Further analysis using COACH and COFACTOR tools on the ITASSER platform revealed an adenosine 5-diphosphate ligand-binding site with several potential enzyme active site residues in these regions ( Table 3).

By multiple sequence alignment of PI4Kβ protein from the different *plasmodium* species, we found a conserved region starting position 1254 to 1552 of the PI4Kβ. Further interrogation using the MOTIF finder traced three plausible motifs. These are PI3Ka, Peptidase_S26, and two PI3_PI4_kinase motifs. Here, we targeted PI4Kβ from position 1280 to 1540 of the protein by PCR amplification which localizes the conserved ligand-binding site at position 1294 – 1370 and houses the two PI3_PI4_kinase motifs and the catalytic domain. Several chemical inhibitors such as MMV 0048 and KDU691 kill *Plasmodium falciparum* by targeting the conserved region of the PI4Kβ ^59^. Acquisition of mutations within the catalytic site of the PI4Kβ decreases parasite susceptibility to chemical compounds MMV 0048 and KDU691 ^59^, supporting our focus on the 1280 to 1540 position of the protein in this study.

Our MOTIF search on CDPK1 showed 14 predicted motifs: the expected Pkinase, PK_Tyr_Ser_Thr, and Kinase-like motifs. Interestingly, the proteins comprise 19 EF-hand motifs, four EF-hand_1, four EF-hand_6, four EF-hand_8, three EF-hand_5, two EF-hand_1, one EF-hand_4, and one EF-hand_9, suggesting high conservation of the protein. Unsurprisingly EF-hand motifs reside in the most conserved carboxyl-terminal end of the protein and show 90% conservation across the six malaria parasite species, suggesting the important function of the motifs in the CDPK1. The ITASSER query showed ligand-binding motifs at positions 145 – 263 which is within our PCR amplification target region ( Table 3).

### Drug response profiles confirmed the stability of the LMr, AQr, and PQr parasites

**Figure 1.**
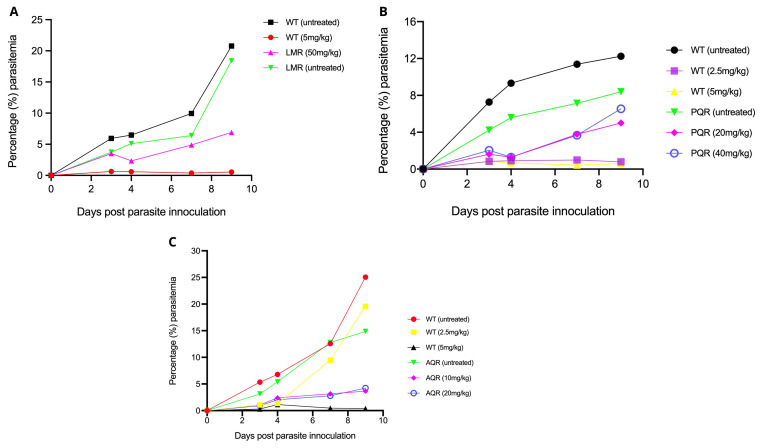
Drug activity profiles as assessed using the 4-day suppressive test. ( **A**) Lumefantrine against the lumefantrine resistant (LMR) and the wild-type drug-sensitive (LMS) *Plasmodium berghei* ANKA parasites at 5mg/kg for wild-type drug-sensitive (WT) and 50mg/kg for LMR. ( **B**) Piperaquine against the piperaquine resistant (PQR) and the WT *Plasmodium berghei* ANKA parasites at 2.5mg/kg and 5mg/kg for the PQS and 20mg/kg and 40mg/kg for the PQR. ( **C**) Amodiaquine against amodiaquine resistant (AQR) and the WT *Plasmodium berghei* ANKA parasites at 2.5mg/kg and 5mg/kg for the AQS and 10mg/kg and 20mg/kg for the AQR. Percentage (%) parasite densities were monitored for a total of nine days post parasite inoculation and five days after cessation of drug treatment.

We first generated baseline drug susceptibility profiles of the LMr, PQr, and AQr parasites against respective drugs for which each line was initially selected. As expected, the untreated LMr or WT grew normally, with parasitemia rising to above 5% by day 4-post parasite inoculation. Submission of WT 5mg/kg cleared the parasite to undetectable levels by microscopy or less than 0.01% parasitemia. LM at 50mg/kg failed to kill the parasite suggesting retention of a stable resistant phenotype. The administration of 2.5mg/kg of AQ or PQ allowed modest growth of the WT parasites to an average parasitemia of 1% throughout the four days of drug administration. However, the WT parasites rose drastically to 9.46% by day 7, three days after cessation of the drug treatment. The WT parasite remained suppressed by 2.5mg/kg of PQ across the entire follow-up period of 9 days post parasite infection, with the highest parasitemia yielding 0.99%. AQ or PQ at 5mg/kg suppressed the WT parasites to below 0.5%, confirming the expected susceptibility of the WT parasites ( Figure 1). We extended our investigation to PQr parasites by submitting them to 20mg/kg or 40mg/kg of PQ; our results show a general consistent rise in parasite densities despite the continuous drug pressure from day 0 to day 3-post parasite inoculation, with an expected sharp rise in parasitemia as recorded on day 7 and day 9-post parasite infection. AQr parasite yielded a steady increase in growth despite administration of 10 or 20 mg/kg ( Figure 1). We found no significant distinction between AQr responses to 10 and 20 mg/kg, which could be attributed to varying AQ/DEAQ metabolism. Overall, the drug response profile shows the susceptibility of the WT to LM, PQ, and AQ. At the same time, their respective resistant parasites had retained resistance phenotypes, suggesting that the resistant mechanisms were encoded within the parasite's genome.

### Mutations in DAGK, PANK1, and PI4Kβ in the amodiaquine resistant parasites

**Figure 2.**
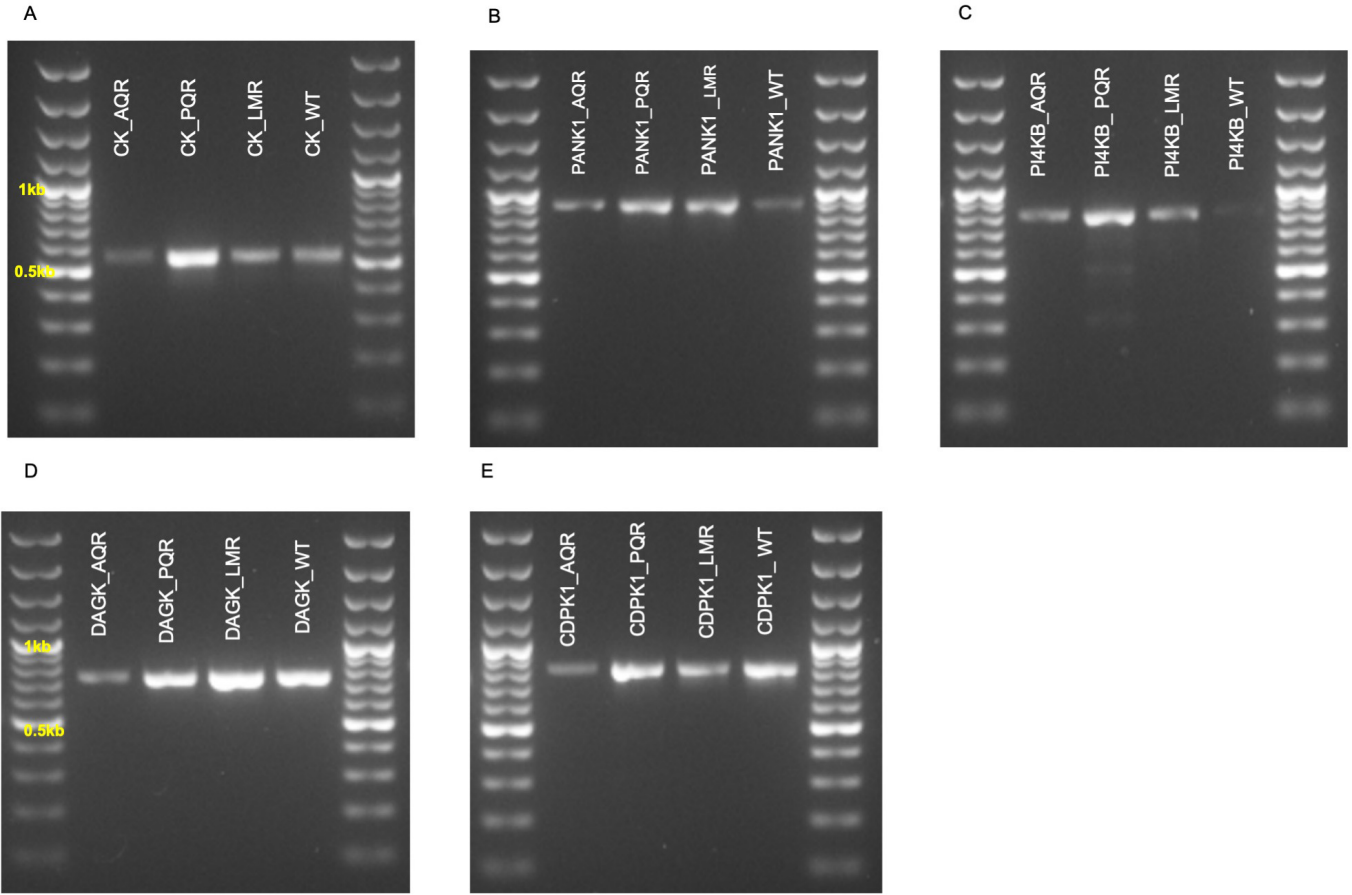
Gel electrophoresis profiles of PCR products ( **A**) choline kinase (CK) ( **B**) pantothenate kinase 1 (PANK1) ( **C**) phosphatidylinositol 4-kinase (PI4KB) ( **D**) diacylglycerol kinase (DAGK) and ( **E**) calcium-dependent protein kinase 1 as amplified from piperaquine resistant (PQR), lumefantrine resistant (LMR), amodiaquine resistant (AQR), and the wild-type drug-sensitive (WT) parasites.

To investigate possible SNPs in the kinases, target regions in *ck*, *pank1*, *dagk*, *pi4kβ*, and *cdpk1* genes were PCR amplified, and the expected PCR products were obtained ( Figure 2). Optimized PCR amplification conditions of the PCR experiments are shown in Table 4. Analysis of the PCR amplified, and sequenced motifs revealed that only DAGK, PANK1, and PI4Kβ possessed nonsynonymous mutations. Surprisingly, the mutations occurred only in the AQr parasites ( Table 5). Parasites mechanically passed for several generations may acquire mutations independent of the drug pressure ^60^. The WT parasites passaged a similar number of mechanical passages and did not acquire the mutations thus, we rule out the possibility of long-term mechanical passages inducing the nonsynonymous mutations observed in DAGK, PANK1, and PI4Kβ. We have also interrogated LMr and PQr parasites selected using a similar protocol but failed to select mutations in the DAGK, PANK1, and PI4Kβ; therefore, we argue that the mutations observed in AQr are derived from AQ pressure. This suggests that AQ or its long-acting metabolite, DEAQ, may submit selective pressure on PANK1, DAGK, and PI4Kβ proteins. We thus propose that submitting AQ pressure on *Plasmodium berghei* ANKA is accompanied by the acquisition of mutation in PANK1, DAGK, and PI4Kβ.

**Table 4.** Optimized condition for PCR amplification of *Plasmodium berghei* choline kinase ( *Pbck*), *Plasmodium berghei* pantothenate kinase 1 ( *Pbpank1*), *Plasmodium berghei* phosphatidylinositol 4-kinase beta ( *Pbpi4kβ*), *Plasmodium berghei* diacylglycerol kinase, putative ( *Pbdagk*), and *Plasmodium berghei* calcium-dependent protein kinase 1 ( *Pbcdpk1*) genes using the Q5 High Fidelity 5x PCR Master Mix (New England Biolabs™).

PCR profiles	Optimized Temperature (°C) /Time (sec/min)	
	*1. Pbck* *2. Pbpi4kβ* *3. Pbdagk* *4. Pbcdpk1*	*Pbpank1*
Initial denaturation	98°C, 10 sec	98°C, 10 sec
Denaturation	98°C, 5 sec	98°C, 5 sec
Annealing Temperature	58°C, 10 sec	59°C, 10 sec
Elongation	72°C, 30 Sec	72°C, 30 Sec
Primer (Forward & reverse)	1.0 pmol/µl each	1.0 pmol/µl each
Cycles	30	30
Final elongation	72°C, 2 min	72°C, 2 min

**Table 5.** A summary of the location of mutations in choline kinase (CK), pantothenate kinase 1 (PANK1) phosphatidylinositol 4-kinase (PI4KB), diacylglycerol kinase (DAGK), and, calcium-dependent protein kinase 1(CDPK1) following multiple sequence alignment using CLUSTAL-Omega of the sequenced DNA ( https://www.ebi.ac.uk/Tools/msa/clustalo/) and the translated DNA with the edited mutations in the genes ( https://www.ebi.ac.uk/Tools/st/emboss_transeq/).

Name of the protein/Accession No	Nucleotide change in the targeted coding sequence			Amino acid change in the protein		
	AQR	PQR	LMR	AQR	PQR	LMR
Choline kinase (CK)/ PBANKA_1040100	-	-	-	-	-	-
Pantothenate kinase 1 (PANK1)/ PBANKA_ 1022600	A1180C	-	-	N394H, Asn→His	-	-
Phosphatidylinositol 4-kinase beta (PI4KB)/ PBANKA_1109400	G4096A T4101G T4180A G4267A	-	-	D1366N, Asp→Asn S1367R, Ser→Arg Y1394N, Tyr→ Asn D1423N, Asp→Asn	-	-
Diacylglycerol kinase, putative (DAGK)/ PBANKA_1334600	A809G A875G	-	-	K270R, Lys→Arg K292R, Lys→Arg	-	-
Calcium-dependent protein kinase 1 (CDPK1)/ PBANKA_0314200	-	-	-	-	-	-

**Figure 3.**
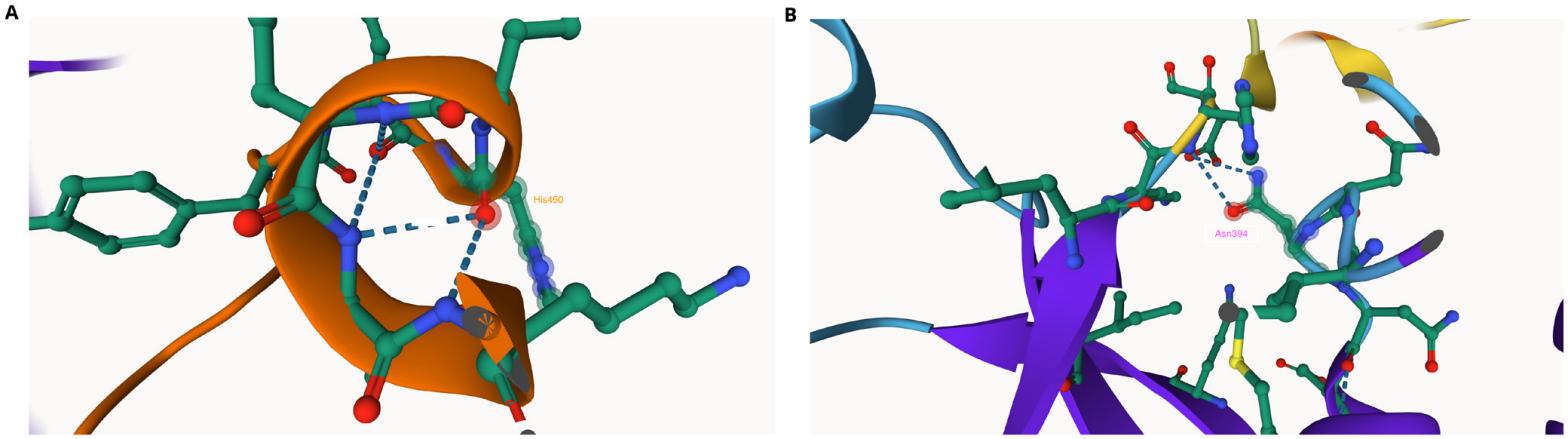
A section of pantothenate kinase 1 (PANK1) as predicted using AlphaFold ( **A**) The linkage of Asn394 with His and Asn in the wildtype protein. ( **B**) The mutation Asn394His abolishes the linkage.

We found one nonsynonymous mutation Asn394His in PANK1 in the AQr parasites ( Figure 3), but LMr and PQr parasites did not have any mutations in PANK1 ( Table 5). We then used AlphaFold and ITASSER tools to predict the impact of the mutation on PANK1 protein, and we show Asn394 interacts with His450 ( Figure 3A, 3B). The Asn394His mutation eliminates the Asn394 – His450 interaction suggesting a possible alteration of protein structure. Histidine has an imidazole side chain, which has a pKa of 6.8, the pH of the cytoplasm ^61^. As a result, small shifts in cellular pH may change the charge of histidine side chains between acid and basic pH. The activities of many proteins are modulated by pH through the protonation of histidine side chains. Asparagine is uncharged but has polar amide groups with extensive hydrogen-bonding capacities. As a result, slight shifts in cellular pH may alter the charge of histidine side chains ^61^. Although Asn and His are polar amino acids, Asn is neutral while His is a basic amino acid, suggesting alteration of the hydrophilicity of the side chain. Also, based on protein structure analysis using AlphaFold of the AQr mutant structure, the amino acid change at position 394 resulted in the introduction of a hydrogen bond and a new interaction with Glu, an interaction illustrated as (394|O-Glu 398|N); this is due to the presence of amide groups with extensive hydrogen-bonding capacities.

Within the targeted motifs of the CK and CDPK1, we found no mutation in AQr, LMr, or PQr resistant parasites ( Table 5). A mutation that impacts a significant effect on phenotype may occur within a function motif of the protein. Here we interrogated an essential functional motif of the CK and CDKP1 protein; thus, our results have two implications: First, LM, PQ, or AQ do not exert selective pressure on CK and CDPK1, therefore are not associated with the resistant phenotypes. Second, CK and CDPK1 are essential for the growth of the asexual blood-stage parasites; thus, the acquisition of a mutation at the functional motif may be toxic for parasites. The parasites likely possess mutations in other protein sections. Whole gene sequencing or whole-genome sequence of the parasites may reveal changes in other functional regions of the protein. Several studies have interrogated the possibility of motif's druggability since significant motifs are composed of the regulatory interplays between phosphorylation and proteolysis ^62, 63^. These studies discovered that motifs are significantly enriched with drug targets, suggesting the possibility of exploring these conserved motifs as potential drug targets ^63^ and thus likely mediators of drug responses. Here we only investigated the highly likely motifs of the CK and CDPK1. Therefore, mutation acquisition in other sections of the proteins may only have subtle changes in altering drug susceptibility.

**Figure 4.**
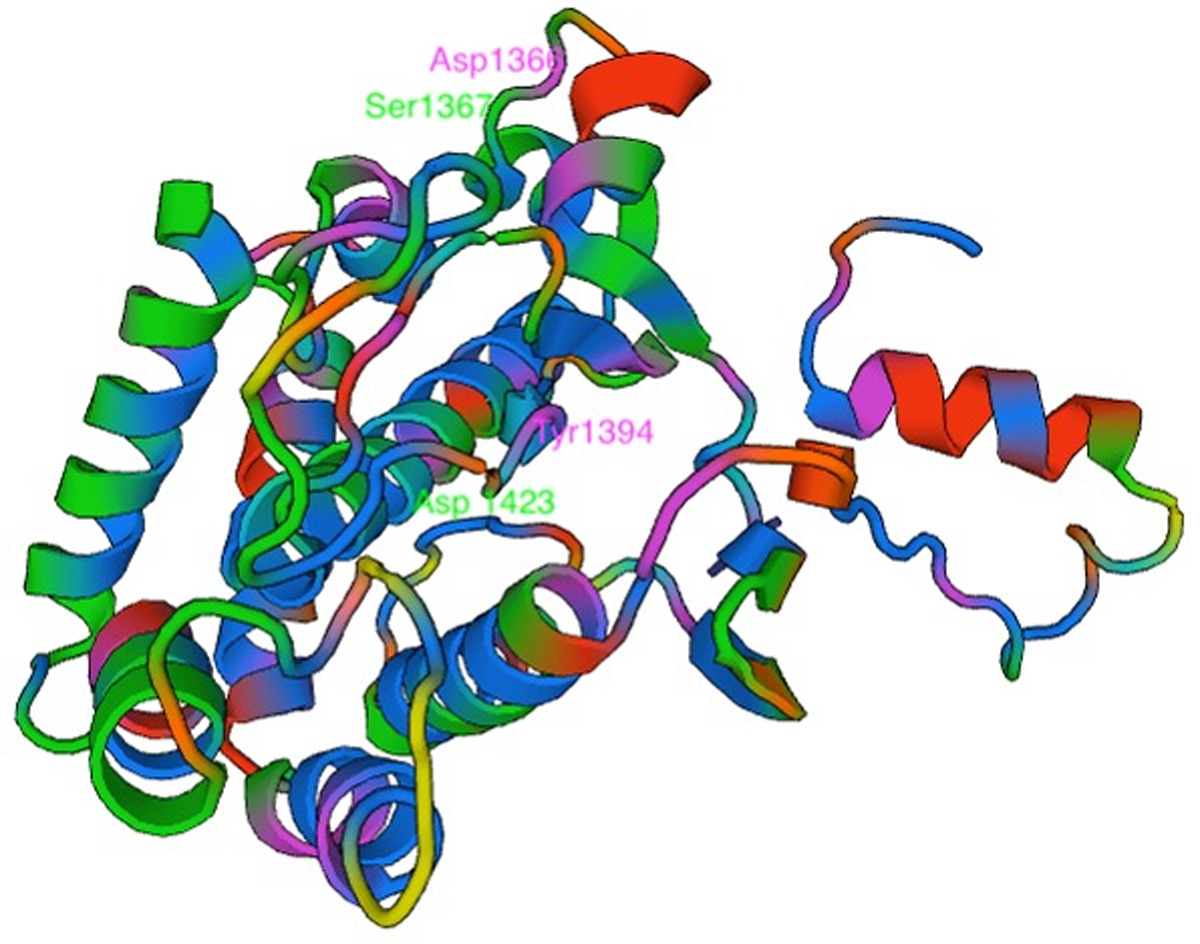
Phosphatidylinositol-4 kinase (PI4Kβ) as predicted using the Swiss Model showing the four mutations Asp1366, Ser1367, Tyr1394, and Asp 1423 acquired in the amodiaquine-resistant parasites.

We then probed PI4K and identified four nonsynonymous SNPs in the AQr parasite and none in PQr and LMr parasites. These are Asn1366Asp, Ser1367Arg, Tyr1394Asn, and Tyr1423Asn ( Table 5). I-TASSER results of PI4Kβ wild type and AQr show a new ligand-binding site for trifluoromagnesate (MGF) at Asp1451Asn. Asp has a carboxylic acid side chain that forms ionic bonds and can also function as hydrogen bond acceptors. These changes might alter the binding efficiency and the protein function. Many proteins that bind metal ions for structural or functional purposes possess metal-binding sites containing aspartate ^64^. Asn has amide R groups, and the amino group (NH _2_) functions as a hydrogen bond donor. I-TASSER also predicted the modified protein structure due to the acquired mutations, further corroborated by AlphaFold protein structure prediction ( Figure 4). AlphaFold prediction on amino acid interactions of the mutated protein showed that the interaction at position 1366 of the protein between Asp and Lys (1366|O-LYS1360|NZ) was retained despite the amino acid change. At position 1367Arg, the mutant protein obtained a new amino acid interaction Thr, illustrated as (1367|0-THR 1365|OG1), and lost two other amino acid interactions (1367|O-ILE 1371|N: 1367|OG-THR 1370|N) but retained (1367|O-LYS 1365|OG1). On the other hand, 1451Asn resulted in two new amino acid interactions between Asn and Gly and Asn and Arg; (1451|OD1-GLY 1453|N: 1451|O-ARG 1434|NH _2_) ( Figure 4). The changes in the interaction of the amino acids and protein structure changes suggest a possible interference with protein function. The role of these changes in mediating AQ resistance requires further investigation.

**Figure 5.**
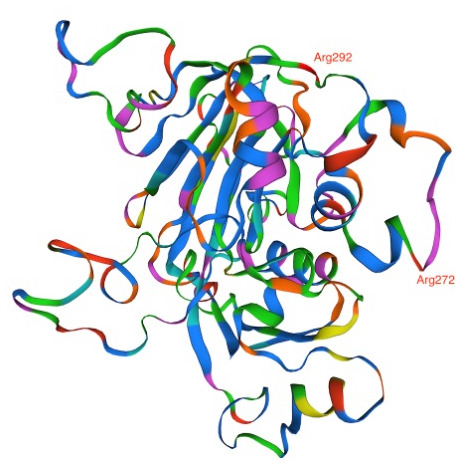
Diacylglycerol kinase (DAGK) as predicted using the Swiss Model showing the two Arg272 and Arg292 mutations acquired by the amodiaquine-resistant parasites.

Analysis of DAGK sequence illustrated amino acid change; Lys270Arg and Lys292Arg in the AQr parasites ( Figure 5). Arginine and lysine are basic amino acids and did not attract changes in new amino acid interactions as predicted by AlphaFold (270|O-ASN 276|ND2 and 292|N-SER 290|OG). Lysine and arginine have an overall charge of +1 at physiological pH. The guanidino group in arginine's side chain is the most basic of all R groups; this led to losing one hydrogen bond at position 270|N-ASN276|OD1. Based on AlphaFold, Lys -> Arg change resulted in modifying the secondary structure of the DAGK protein. To determine the effect of these mutations on the protein structure and specifically on the ligand and enzyme binding sites, we queried the mutant DAGK protein in ITASSER. This revealed different ligand binding sites on the mutant DAGK protein with altered enzyme active sites, such as an ADP ligand-binding site.

### Differential gene expression of PANK1, PI4Kβ, and DAGK genes in AQr parasite lines

**Figure 6.**
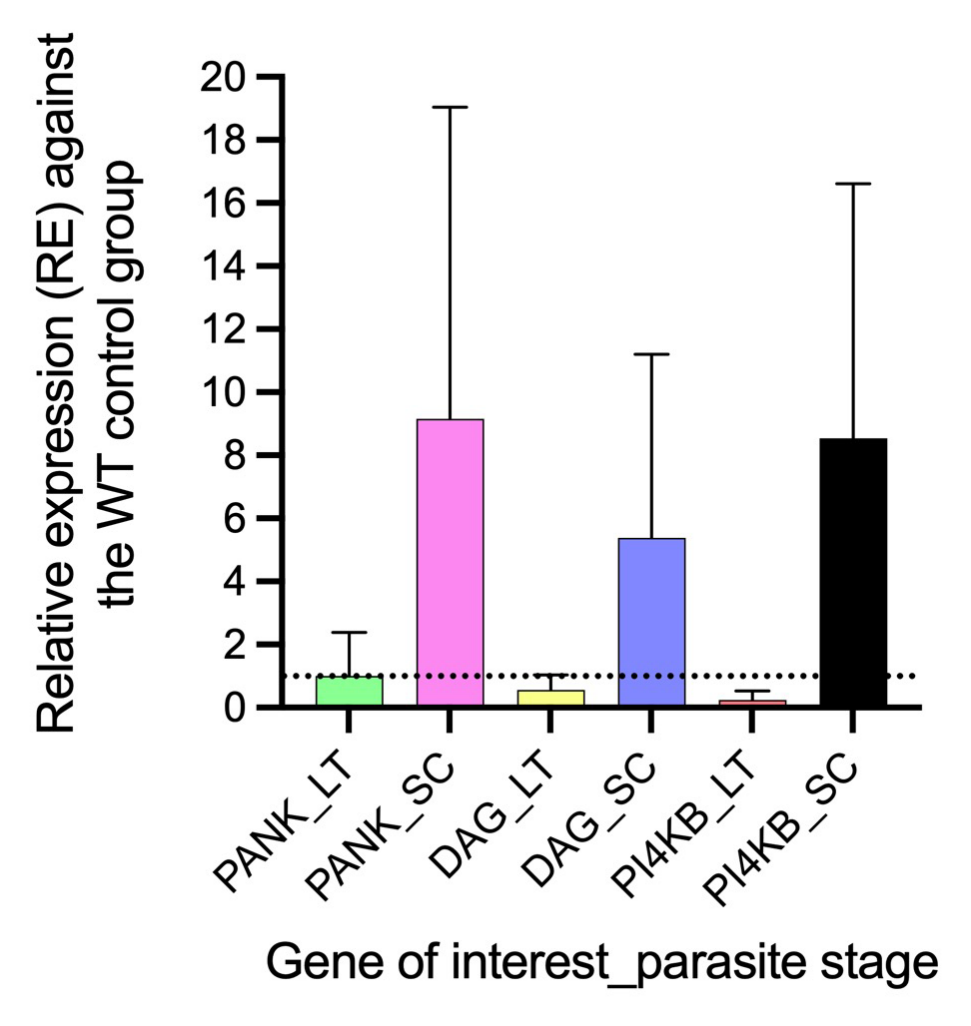
The relative gene expression of pantothenate kinase (pank1), diacylglycerol kinase (dagk), and phosphatidylinositol 4-kinase (pi4kβ) at late trophozoite (LT) and schizonts (SC) stages in the amodiaquine-resistant parasites relative to the drug-sensitive control parasites. The differential expression from a mean of at least three independent experiments was significantly upregulated for pank1, dagk, and pi4kβ at the schizont stage and downregulated at the trophozoite stage.

Parasites may adapt to drug pressure through upregulation or downregulation of essential drug targets or drug modifying proteins ^65^. To evaluate whether gene expression changes accompanied the mutation's acquisition, we measured the mRNA amount of PANK1, PI4Kβ, and DAGK. We argued that maximal differential expression would be expected at the trophozoites and early schizonts, the stages at which AQ and DEAQ are predicted to exert maximal action ^66^. We reveal a differential gene expression in PANK1, PI4Kβ, and DAGK at trophozoites and early schizont stages between AQr and drug-sensitive parasites ^42^. All three genes were significantly upregulated in the schizont stages and downregulated in the trophozoite stages ( Figure 6). The expression level of PANK1 at the trophozoite stages was not significantly altered despite showing marginal downregulation by 1.3-fold ( *p<0.4815*) but was significantly upregulated by 7.01-fold ( *p<0.001*) during the schizont stages. In a similar trend, DAGK showed 1.2-fold downregulation at the trophozoite ( *p<0.086)* and 5.4-fold ( *p<0.001)* upregulation at the schizonts. Interestingly, PI4Kβ had the highest differential gene expression yielding a 7.1-fold ( *p<0.001)* downregulation at the trophozoite and 9.0-fold ( *p<0.0035)* upregulation at the schizont stages. The impact of the differential expression of PANK1, PI4Kβ, and DAGK genes in mediating drug responses requires validation.

## Conclusion

**Figure 7.**
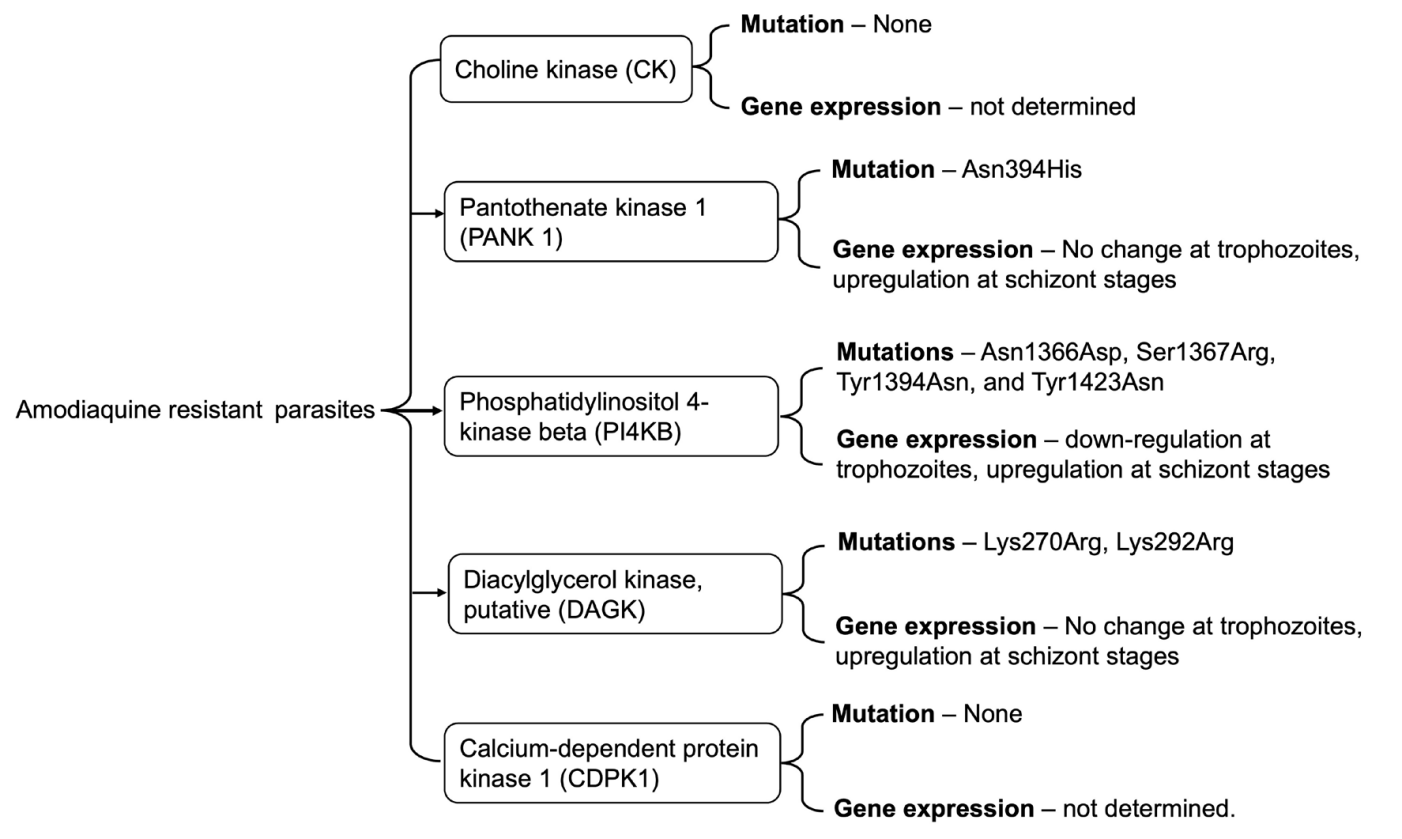
A summary of key findings of associated mutations and gene expression profiles of Choline kinase, Pantothenate kinase 1, Phosphatidylinositol 4-kinase beta, Diacylglycerol kinase, and Calcium-dependent protein kinase 1 in amodiaquine-resistant *Plasmodium berghei* ANKA.

Here, we show the selection of nonsynonymous mutations in malaria parasites' druggable kinases: the PANK1, DAGK, and PI4Kβ ( Figure 7). The mutation occurred within or close to ligand binding sites or predicted active sites. Selectively acquiring these mutations in AQr resistant and not in the LMr or PQr resistant parasites may signify proteins under AQ or metabolite DEAQ pressure. We further show downregulation of PANK1, DAGK, and PI4Kβ in the trophozoites but upregulation at the schizonts stages in the AQr parasites. Future studies employing reverse genetics techniques to determine and validate the impact of the mutations in modifying drug susceptibility may reveal important insights into understanding the mechanisms of AQ resistance in malaria parasites.

## Data Availability

Open science framework: ‘Amodiaquine drug pressure selects nonsynonymous mutation in pantothenate kinase 1, diacylglycerol kinase, and phosphatidylinositol-4 kinase in *Plasmodium berghei* ANKA’ https://doi.org/10.17605/OSF.IO/WHDT5 ^42^. This project contains the following underlying data: Amodiaquine_baseline_profile.xlsx (Amodiaquine-resistant and wild-type parasite responses to amodiaquine drug). CDPK1_DNA_alignment.rtf (Multiple sequence alignment of *cdpk1* gene sequence data from LMr, PQr, and AQr). cdpk1 UVP02160July262022.tif (gel photo of cdkp1 gene amplification results) chk and pank1 gel key.docx (description key for chk and pank1 gene amplification results) chk and pank1 UVP02167July262022.tif (gel photo of chk and pank1 gene amplification results) CK_DNA_alignment.rtf (Multiple sequence alignment of ck gene sequence data from LMr, PQr, and AQr). cpdk1 gel key.docx (description key for cdpk1 gene amplification results) DAGK_DNA_alignment.rtf (Multiple sequence alignment of *dagk* gene sequence data from LMr, PQr, and AQr). Lumefantrine_baseline_profile.xlsx (Lumefantrine-resistant and wild-type parasite responses to lumefantrine drug). PANK1_DNA_alignment.rtf (Multiple sequence alignment of *pank1* gene sequence data from LMr, PQr, and AQr). PI4Kβ _DNA_alignment.rtf (Multiple sequence alignment of *pi4k*β gene sequence data from LMr, PQr, and AQr). pi4kb and dagk jean GEL key.docx (description key for pi4kb and dagk gene amplification results) pi4kb and dagk UVP02161July262022.tif (gel photo of pi4kb and dagk gene amplification results) Piperaquine_baseline_profile.xlsx (Piperaquine-resistant and wild-type parasite responses to piperaquine drug). qPCR_relative gene_expression_raw_data.xlsx (Cq values for *Pbdagk*, *Pbpank1*, and *Pbpi4kβ*). Figure S1: A flow chart summary of selected protein kinases as mediators of drug target, action, locatlization and resistance. Open Science framework: ARRIVE checklist for ‘Amodiaquine drug pressure selects nonsynonymous mutation in pantothenate kinase 1, diacylglycerol kinase, and phosphatidylinositol-4 kinase in *Plasmodium berghei* ANKA’ https://doi.org/10.17605/OSF.IO/WHDT5 ^42^. Data are available under the terms of the Creative Commons Zero "No rights reserved" data waiver (CC0 1.0 Public domain dedication).

## Accession numbers

Gene: calcium-dependent protein kinase 1 [ Plasmodium berghei ANKA]. Accession number: PBANKA_0314200. https://www.ncbi.nlm.nih.gov/gene/?term=PBANKA_0314200

Gene: choline kinase [Plasmodium berghei ANKA]. Accession number: PBANKA_1040100. https://www.ncbi.nlm.nih.gov/gene/?term=PBANKA_1040100

Gene: pantothenate kinase 1, putative [ Plasmodium berghei ANKA]. Accession number: PBANKA_1022600. https://www.ncbi.nlm.nih.gov/gene/55150156.

Gene: diacylglycerol kinase, putative [ Plasmodium berghei ANKA]. Accession number: PBANKA_1334600. https://www.ncbi.nlm.nih.gov/gene/?term=PBANKA_1334600

Gene: phosphatidylinositol 4-kinase, putative [ Plasmodium berghei ANKA]. Accession number: PBANKA_1109400. https://www.ncbi.nlm.nih.gov/gene/?term=PBANKA_1109400
