# Supplementary material for: Amodiaquine drug pressure selects nonsynonymous mutations in pantothenate kinase 1, diacylglycerol kinase, and phosphatidylinositol-4 kinase in Plasmodium berghei ANKA
Source: Open Res Afr. 2023 Oct 19;5:28. Originally published 2022 Aug 19. [Version 3] doi: 10.12688/openresafrica.13436.3 (PMC11195610; doi:10.12688/openresafrica.13436.3)
Supplement: Peer review history

# Reviewer report: Reviewer response for version 3

**DOI:** 10.21956/openresafrica.15632.r31974

**Published:** 2025-01-15

**Authors:** George Ghartey-Kwansah

**Affiliations:** 1 University of Cape Coast, Cape Coast, Central, Ghana

Generally, the manuscript is well written with minor corrections. Example, CDPK1 is sometimes written as CDKP1. Kindly do the needful.

**There are however few question to be asked:**

- What informed the selection of these kinases?
- Why did the authors single out CDPK1 out of the CDPK family?
- Why were the authors silent on synonymous mutation? It must be noted that synonymous mutation can influence gene expression levels as well as protein structure. 

Is the work clearly and accurately presented and does it cite the current literature?

Yes

If applicable, is the statistical analysis and its interpretation appropriate?

Yes

Are all the source data underlying the results available to ensure full reproducibility?

Yes

Is the study design appropriate and is the work technically sound?

Yes

Are the conclusions drawn adequately supported by the results?

Yes

Are sufficient details of methods and analysis provided to allow replication by others?

Yes

Reviewer Expertise:

Using both  dry and wet lab techniques to understand NTDs, Malaria, Schistosomiasis etc

I confirm that I have read this submission and believe that I have an appropriate level of expertise to confirm that it is of an acceptable scientific standard.

# Reviewer report: Reviewer response for version 3

**DOI:** 10.21956/openresafrica.15632.r31999

**Published:** 2025-01-15

**Authors:** Haddijatou Mbye

**Affiliations:** 1 University of Cambridge, Cambridge, UK

The manuscript titled “Amodiaquine drug pressure selects nonsynonymous mutations in pantothenate kinase 1, diacylglycerol kinase, and phosphatidylinositol-4 kinase in *Plasmodium berghei *ANKA” investigates the impact of drug pressure from three key ACT partner drugs on the genetic makeup of *Plasmodium berghei *ANKA. The study focuses on the potential role of key protein kinases in mediating drug resistance.

 This study is particularly interesting because it investigates resistance mechanisms against long-acting partner drugs in ACTs, a cornerstone of malaria treatment. By focusing on kinases, which are critical regulators of cellular processes, the research opens up new avenues for understanding malaria drug resistance at a molecular level.

**Minor points to address:**

**Introduction:**

- In the introduction, the authors reference malaria statistics from the WHO from 2020. The most recent World Malaria Report by the WHO (published in 2023) provides updated figures on malaria cases, deaths and global trends. For accuracy and relevance, these figures should be updated to reflect the latest statistics.
- The authors should clarify the difference between resistance mediated by drug metabolites (DEAQ) and AQ itself.
- The sentence *"In Plasmodium, protein kinase C involved in the growth, maturation, and differentiation of all the asexual blood stages can be inhibited by CQ, but only in the CQ-sensitive Plasmodium falciparum, suggesting a possible association between the kinase with CQ susceptibility in the CQ resistant P. falciparum"* is unclear. It is contradictory to suggest an association between protein kinase C and CQ susceptibility in CQ-resistant parasites while stating it is inhibited only in CQ-sensitive parasites. The authors should reword this sentence to clarify whether protein kinase C is a mediator of CQ susceptibility or resistance.
- The sentence *"Other kinase-based inhibitors that have progressed to human clinical trials are phosphatidylinositol 4-kinase (PI4K), with inhibitor 37 (MMV048)"* is confusing. PI4K is not an inhibitor but a kinase. The authors should reword to clarify that PI4K is the target and MMV048 is the inhibitor molecule that acts on PI4K. I propose rephrasing it to: *"Phosphatidylinositol 4-kinase (PI4K) has been identified as a target for kinase-based inhibitors, such as MMV048, which has progressed to human clinical trials."*
- Replace technical abbreviations like "AQr" with "AQ-resistant" when first introduced and ensure all abbreviations are defined in the text.

**Methods:**

- The sentence *"PCR products were analyzed in 2% agarose gel, purified using the AddBio PCR Purification Kit (addbio™ Cat No 10078), and sequenced based on BigDye v3.1 using a 3730xl sequencer based on BigDye v3.1"* unnecessarily repeats "BigDye v3.1”. Remove the repeated reference to "BigDye v3.1"

**Results and discussion:**

- In the results, the study identifies specific non-synonymous mutations. Authors should discuss whether these mutations were previously reported or novel.
- In some instances, the text does not explicitly direct readers to the relevant figures or tables (e.g., gene expression results in Figure 6). Authors should use phrases like "As shown in Figure X..." to guide readers more effectively.
- The "Position" and "Tm" columns in Tables 1 and 2 lack the appropriate units. For clarity and completeness. Add "(bp)" after "Position" to indicate that the values represent base pairs and add "°C" after "Tm" to specify that the values refer to melting temperature in degrees Celsius.
- The sentence " *Interestingly, the proteins comprise 19 EF-hand motifs, four EF-hand_1, four EF-hand_6, four EF-hand_8, three EF-hand_5, two EF-hand_1, one EF-hand_4, and one EF-hand_9, suggesting high conservation of the protein.*" contains inconsistencies in the enumeration of EF-hand motifs, specifically the repeated mention of "four EF-hand_1" and "two EF-hand_1." Clarify and ensure the total count matches the claimed 19 EF-hand motifs.
- The column heading on Table 3: *"Number of motifs and regions of shared predicted by MOTIF Search"* appears incomplete or grammatically incorrect. Authors should revise the heading for clarity.
- In the discussion, the authors should emphasise the broader implications of these mutations for antimalarial resistance in other *Plasmodium* species.

**Conclusion:**

- Authors should avoid redundancy by summarising points concisely. For example, certain findings such as the stage-specific expression of kinases, are repeated multiple times.

Is the work clearly and accurately presented and does it cite the current literature?

Yes

If applicable, is the statistical analysis and its interpretation appropriate?

Yes

Are all the source data underlying the results available to ensure full reproducibility?

Yes

Is the study design appropriate and is the work technically sound?

Yes

Are the conclusions drawn adequately supported by the results?

Yes

Are sufficient details of methods and analysis provided to allow replication by others?

Yes

Reviewer Expertise:

Malaria, Drug resistance, Genomics, Epigenetics,

I confirm that I have read this submission and believe that I have an appropriate level of expertise to confirm that it is of an acceptable scientific standard.

# Reviewer report: Reviewer response for version 3

**DOI:** 10.21956/openresafrica.15632.r31906

**Published:** 2025-01-13

**Authors:** Nancy Odurowah Duah-Quashie

**Affiliations:** 1 University of Ghana, Accra, Ghana

The authors sought to investigate whether variants in the predicted functional motifs of the protein kinases - choline kinase (CK), pantothenate kinase 1 (PANK1), diacylglycerol kinase (DAGK), and phosphatidylinositol-4 kinase (PI4Kβ), and calcium-dependent protein kinase 1 (CDPK1) – are associated with parasite resistance to the ACT partner drugs Lumefantrine (LM), piperaquine (PQ), and amodiaquine (AQ).

 The authors highlight the rationale of their work by arguing the need to look for unconventional genes at play in the development of antimalarial drug resistance in malaria parasites.

**Comments:**

 1. The authors focused on protein kinases by providing evidence from the existing literature to suggest that they can be implicated in the molecular mechanisms undergirding AQ, LM, and PQ resistance. This crucial argument to the importance of the authors’ work, although logical, barely paints a very convincing premise. Even though the bulk of the introduction is spent on this endeavor, what remains palpably missing is how (even if hypothetically), kinases can mediate AQ, PQ, and LM resistance in malaria parasites given what is known about their purported mechanisms of action. The authors’ concession that "While a link between kinases and AQ, LM, or PQ in *Plasmodium* remains unclear, further insight makes it relevant for the authors to propose a conceptual hypothetical framework detailing how the kinases of interest can cause AQ, PQ, and LM resistance. This might be considered the biggest deficiency of the manuscript.

 2. **The second comment with the manuscript is its abstract:**

 a. The method section of the abstract can be improved by justifying the approach used to interrogate the research question. i.e. why did the authors use *in silico* tools to look first for ligand binding motifs, active sites, and conserved regions and then for NS SNPs in the predicted functional motifs of the genes of interest? It is not clear why mRNA transcripts of the GOI were measured at schizont and trophozoite stages and not in drug resistant and susceptible parasites. We are not told which parasite lines - lab or clinical- and the number of biological and technical replicates used for the wet-lab. The abstract should clearly state that the parasite lines used in the study were drug resistant lines.

 b. The method description in the abstract makes it difficult to appreciate the scope of the work done and the results shared in the abstract. E.g. It is difficult to grasp the intended import of the non-synonymous (NS) variants listed in the kinases investigated. It is difficult to see the meaning of the observation reported that mRNA of some kinases of interest were upregulated in the schizont stage. It would be clearer if it was clearly stated whether this observation was in a AQ drug resistant line. The use of the adjective "surprisingly" should be taken out.

 c. In the conclusion of the abstract, if the research objective was to interrogate the causal association, if any, between NS variants in kinases of interest and ACT partner drug resistance, the conclusion as stated does not highlight this convincingly.

 The use of the phrase "selective acquisition of mutations" suggests a longitudinal drug selection of resistant parasites with characterization of variants at selected time points on the temporal axis. This was not done in this study. It might improve the clarity of the abstract if this phrase is revised.

 The fact that most of these concerns were assuaged by reading the entire method, results and discussion sections of the manuscripts speaks to the fact that the abstract in its current form is inadequately written and must be improved.

  

1. The method fails to disclose at the onset how the authors controlled for the possibility that the variants reported might have been due to random mutations from the 40 passages and not AQ pressure. Given that the AQr lines used were generated by 40 passages under drug pressure, it would have been ideal to use, as the wild type controls, the same drug free controls with 40 passages instead of “ **Two parasite reference lines of *P. berghei* ANKA (the MRA-865 and MRA-868 reference lines obtained from MR4, ATCC® Manassas, Virginia) were used as the drug-sensitive…**”.

“ **The WT parasites passaged a similar number of mechanical passages and did not acquire the mutations thus, we rule out the possibility of long-term mechanical passages inducing the nonsynonymous mutations observed in DAGK, PANK1, and PI4Kβ. We have also interrogated LMr and PQr parasites selected using a similar protocol but failed to select mutations in the DAGK,PANK1, and PI4Kβ; therefore, we argue that the mutations observed in AQr are derived from AQ pressure**.” This is crucial to accepting the crux of the authors' findings in the data presented. Can the authors show these results? Perhaps Sanger sequences in the supplementary as evidence for these statements?

Is the work clearly and accurately presented and does it cite the current literature?

Partly

If applicable, is the statistical analysis and its interpretation appropriate?

Partly

Are all the source data underlying the results available to ensure full reproducibility?

Partly

Is the study design appropriate and is the work technically sound?

Partly

Are the conclusions drawn adequately supported by the results?

Partly

Are sufficient details of methods and analysis provided to allow replication by others?

Partly

Reviewer Expertise:

Antimalarial Drug resistance

I confirm that I have read this submission and believe that I have an appropriate level of expertise to confirm that it is of an acceptable scientific standard, however I have significant reservations, as outlined above.

# Reviewer report: Reviewer response for version 3

**DOI:** 10.21956/openresafrica.15632.r31895

**Published:** 2024-12-21

**Authors:** Dhaneswar Prusty

**Affiliations:** 1 Central University of Rajasthan, Ajmer, India

The well-designed manuscript may offer valuable information for understanding lumefantrine, piperaquine and amodiaquine resistance.

  

1. It is recommended that the quality of the manuscript's literature needs to be enhanced by rectifying grammatical and punctuation errors and reducing unnecessary descriptions.
2. For a mechanistic understanding, comparing docking scores of lumefantrine, piperaquine, and amodiaquine with the wild type vs. identified mutants of target kinases is recommended.
3. The result of Figure 6 is not statistically significant; quantitative PCR analysis is recommended for the expression profile analysis of the studied kinases in  WT vs resistant parasites.
4. It is recommended to strengthen the discussion by establishing the connection between the findings *P. berghei ANKA* and their potential application to *P. falciparum* or other malaria species.

Is the work clearly and accurately presented and does it cite the current literature?

Yes

If applicable, is the statistical analysis and its interpretation appropriate?

No

Are all the source data underlying the results available to ensure full reproducibility?

Yes

Is the study design appropriate and is the work technically sound?

Yes

Are the conclusions drawn adequately supported by the results?

Yes

Are sufficient details of methods and analysis provided to allow replication by others?

Yes

Reviewer Expertise:

Antimalaria drug discovery

I confirm that I have read this submission and believe that I have an appropriate level of expertise to confirm that it is of an acceptable scientific standard, however I have significant reservations, as outlined above.

# Reviewer report: Reviewer response for version 3

**DOI:** 10.21956/openresafrica.15632.r31091

**Published:** 2024-07-04

**Authors:** Aditi Mukherjee, Apabrita Ayan Das

**Affiliations:** 1 Immunology and Infectious Diseases, Harvard T.. Chan School of Public Health, Boston, Massachusetts, USA; 2 Cardiovascular Medicine, Brigham and Women's Hospital, Boston, Massachusetts, USA; 3 Harvard Medical School, Boston, USA

**Minor comments:**

**Introduction**

 The introduction section is well written and concise. I would suggest that the full form of CQ (chloroquine) should be provided first, followed by referring to it as CQ (in the second paragraph).

**Methods**

1. Either use the unit g or RPM.
2. The concentration of DNA used for PCR reactions is missing. It’s written as 2µl of pg DNA.

**Results and Discussion**

1. In the 2 ^nd^ paragraph, mention what’s ABS?
2. Fig 1A: rearrange the labeling. LMR (untreated) should come first, followed by LMR (50mg/kg).

**Major comments:**

 In their RNA extraction protocol, authors mentioned that they dried RNA on a hot plate for 3-5min. This seems very absurd as this is a normalized protocol for drying DNA, but RNA is highly degradable. Did they measure the ratio of A260/A280? This seems to be a major concern because in Fig 6, the standard deviation is way too high. As the number of independent experiments is less, the difference may not be conclusive. Although the overall values are statistically significant, the authors might want to repeat this experiment to increase the number of independent experiments which might lead to a firm conclusion. Also, please mention the statistical significance of the data in the figure itself.

 Authors have used alpha fold and ITASSER to predict the structure of the kinases and MOTIF finder to find the motif.  However, from the ribbon model it is hard to determine the tertiary structure of the proteins and hence the active motifs. Authors may replace/add the ribbon models with tertiary structures (sphere models). Also, authors might consider verifying the results of motif finder with other freely available software and energy minimization algorithms.

Is the work clearly and accurately presented and does it cite the current literature?

Yes

If applicable, is the statistical analysis and its interpretation appropriate?

Partly

Are all the source data underlying the results available to ensure full reproducibility?

Yes

Is the study design appropriate and is the work technically sound?

Partly

Are the conclusions drawn adequately supported by the results?

Partly

Are sufficient details of methods and analysis provided to allow replication by others?

Yes

Reviewer Expertise:

We confirm that we have read this submission and certain that we have an appropriate level of expertise to confirm that it is of an acceptable scientific standard, however we have significant reservations, as outlined above.

We confirm that we have read this submission and believe that we have an appropriate level of expertise to confirm that it is of an acceptable scientific standard, however we have significant reservations, as outlined above.

# Reviewer report: Reviewer response for version 3

**DOI:** 10.21956/openresafrica.15632.r31253

**Published:** 2024-06-18

**Authors:** Jyotsna Chawla

**Affiliations:** 1 Nova Southeastern University, Florida, USA

**Amodiaquine drug pressure selects nonsynonymous mutations in pantothenate kinase 1, diacylglycerol kinase, and phosphatidylinositol-4 kinase in Plasmodium berghei ANKA** [version 3; peer review: 1 approved, 1 approved with reservations]

**Summary:**

 The research article titled "Amodiaquine drug pressure selects nonsynonymous mutations in pantothenate kinase 1, diacylglycerol kinase, and phosphatidylinositol-4 kinase in Plasmodium berghei ANKA" by Jean Chepngetich and colleagues, investigates the association of protein kinases with resistance to amodiaquine, lumefantrine, and piperaquine in Plasmodium berghei ANKA. Using in silico tools, they identified conserved motifs and ligand binding sites in choline kinase, pantothenate kinase 1, diacylglycerol kinase, phosphatidylinositol 4-kinase beta, and calcium-dependent protein kinase 1. They found nonsynonymous mutations in pantothenate kinase 1, diacylglycerol kinase, and phosphatidylinositol 4-kinase beta only in the amodiaquine-resistant parasites. The mutations occurred within or near ligand binding sites or active sites. The expression of pantothenate kinase 1, diacylglycerol kinase, and phosphatidylinositol 4-kinase beta was downregulated in trophozoites but upregulated in schizonts of the amodiaquine-resistant parasites. The research concludes that the selective acquisition of mutations and differential gene expression in AQ-resistant parasites may indicate proteins under AQ pressure.

 Major concerns:

1. The researchers concluded that the selective mutations and differential gene expression in the amodiaquine-resistant parasites may indicate proteins under amodiaquine pressure. However, this assumption is not supported by direct experimental evidence that would confirm the association between the identified mutations and amodiaquine resistance. If reverse genetics is out of the scope of this paper, perhaps the authors could use gene network analysis or identify genes that interact with gene partners that have already been associated with AQ resistance. I would recommend mining PlasmoDB to identify these gene partners.
2. Alternatively, the authors could check if P. *falciparum* orthologs of the kinases have been associated with AQ selection/pressure.

Minor concerns:

1. Data Inclusion: Incorporate data comparing the viability of drug-sensitive versus drug-resistant parasites. This could involve phenotypic evaluations or measurements of growth replication to support the findings.
2. Figure Improvements: Include missing marker labels in figures, provide a figure illustrating key findings related to PANK1, PI4Kβ, and DAGK, and consider adding a figure to demonstrate the link between protein kinases and drug action/resistance.
3. Strengthen qPCR Data Presentation: Include all necessary information in the qPCR data presentation, such as housekeeping gene data, statistical determinants, and the number of biological replicates.
4. **Figure 2. **Gel electrophoresis profiles of PCR products: The authors could crop the extra ladder in the figure as it is redundant

Is the work clearly and accurately presented and does it cite the current literature?

Yes

If applicable, is the statistical analysis and its interpretation appropriate?

Partly

Are all the source data underlying the results available to ensure full reproducibility?

Yes

Is the study design appropriate and is the work technically sound?

Partly

Are the conclusions drawn adequately supported by the results?

Partly

Are sufficient details of methods and analysis provided to allow replication by others?

Partly

Reviewer Expertise:

Malaria, Genomics, Parasitology, Genetic Screens, Vaccines

I confirm that I have read this submission and believe that I have an appropriate level of expertise to confirm that it is of an acceptable scientific standard, however I have significant reservations, as outlined above.

# Reviewer report: Reviewer response for version 3

**DOI:** 10.21956/openresafrica.15632.r31335

**Published:** 2024-06-08

**Authors:** Dina Coertzen

**Affiliations:** 1 University of Pretoria, Pretoria, South Africa

The manuscript: “Amodiaquine drug pressure selects nonsynonymous mutations in pantothenate kinase 1, diacylglycerol kinase, and phosphatidylinositol-4 kinase in Plasmodium berghei ANKA” evaluated the development of mutations to known ACT partner drugs in various key kinases in *P. berghei *ANKA parasites.

 From a biological perspective this link is interesting as the role of kinases and associated signaling cascades has not been investigated in P. falciparum drug resistant lines. There are some major points that I think the authors need to take into consideration, in order to substantiate their findings.   

 For the introduction section:

1. I would propose a rewrite of the second sentence in the introduction as follows: “Of the six species that infect humans, Plasmodium falciparum is the most virulent, **and is responsible for** majority of deaths.”
2. The sentence “LM is an aryl alcohol drug, predicted to share mechanisms of action with 4-aminoquinoline 9.” Can be moved up after the “Like chloroquine, AQ and PQ are aminoquinoline drugs predicted to exert their action within the parasite digestive vacuole by inhibiting toxic heme polymerization.” sentence. 
3. The authors need to include examples or references to the following statement: “These studies also indicate that the mechanisms of AQ, PQ, and LM may involve other unexplored essential parasite proteins.”
4. I am unsure what the authors mean by the term “protein kinases are drug activators”? Perhaps include a reference to this.
5. Edit the following sentence as suggested: “In Plasmodium species, the protein kinases are drug activators, potential targets for **the** drug metabolites, and mediators of drug resistance,32–34.”
6. I think the bolded number “37” may be a typographical error: “Other kinase based-inhibitors that have progressed to human clinical trials are phosphatidylinositol 4-kinase (PI4K), with inhibitor **37** (MMV048)23,37.”
7. The statement highlighted in point 8, above, can also include the following citation: “Brunschwig C, Lawrence N, Taylor D, Abay E, Njoroge M, Basarab GS, Le Manach C, Paquet T, Cabrera DG, Nchinda AT, de Kock C, Wiesner L, Denti P, Waterson D, Blasco B, Leroy D, Witty MJ, Donini C, Duffy J, Wittlin S, White KL, Charman SA, Jiménez-Díaz MB, Angulo-Barturen I, Herreros E, Gamo FJ, Rochford R, Mancama D, Coetzer TL, van der Watt ME, Reader J, Birkholtz LM, Marsh KC, Solapure SM, Burke JE, McPhail JA, Vanaerschot M, Fidock DA, Fish PV, Siegl P, Smith DA, Wirjanata G, Noviyanti R, Price RN, Marfurt J, Silue KD, Street LJ, Chibale K. UCT943, a Next-Generation Plasmodium falciparum PI4K Inhibitor Preclinical Candidate for the Treatment of Malaria. Antimicrob Agents Chemother. 2018 Aug 27;62(9):e00012-18. doi: 10.1128/AAC.00012-18. Erratum in: Antimicrob Agents Chemother. 2018 Oct 24;62(11): PMID: 29941635; PMCID: PMC6125526.”
8. Edit the following sentence as suggested: “ **Commonly,** G **g**enes and mutations mediating resistance to one class may be associated with cross-resistance to drugs of unrelated chemical classes and modes of action.”
9. Include a ref to the following sentence: “Commonly, genes and mutations mediating resistance to one class may be associated with cross-resistance to drugs of unrelated chemical classes and modes of action.”

Scientific results and methods

1. The observation that nonsynonymous mutations only occurred in AQr parasites is strange. This drug is of the same mechanism of action and chemical class as the PQ (8-aminoquinolines), yet no kinase specific mutations were observed in the PQr line. The authors need to provide some justification as to this observation. This is in line with the following sentence in the manuscript:” We thus propose that submitting AQ pressure on Plasmodium berghei ANKA is accompanied by the acquisition of mutation in PANK1, DAGK, and PI4Kβ.” The authors need to give some reference to any biological consideration that could justify this observation, and why specifically this occurs within a AQr line.
2. Furthermore, the authors showed no justification or validation that mutations are specific to kinases in the drug resistant AQr line. There is no sequencing data of other genes presented in this paper. I think that if the authors want to emphasize the synonymous mutation of the specific kinases they need to show WGS full genomic sequence analysis, for at least a couple of parasites. Or at least include several other genes in the PCR experiment. The authors even state this in the paper: “ The parasites likely possess mutations in other protein sections. Whole gene sequencing or whole-genome sequence of the parasites may reveal changes in other functional regions of the protein.”
3. The use of SI units and scientific nomenclature in the methods section is incorrect. Minutes should be min hour hour/hours are h, rpm’s are not SI units and need to be converted to x *g *(note italics of *g)*, ml and µl should be mL and µL, degrees should be °C, spaces should be included between all SI units.
4. All text the manuscript must be justified.
5. I think the authors needed to include some data as the viability of drug sensitive vs drug resistant parasites, either as a phenotypic evaluation or measurement of growth replication.
6. Most importantly to truly assess the effect of kinase specific mutations, drug shift assays with known kinase inhibitors with and without PQ, AQ and LM needed to be performed. This result is critical to this paper.
7. The following sentence is unnecessary:” At the same time, their respective resistant parasites had retained resistance phenotypes, suggesting that the resistant mechanisms were encoded within the parasite’s genome.” All mutations incurred in parasite drug resistant lines occur on a genomic level.
8. The following sentence needs to be rewritten: “The WT parasites passaged a similar number of mechanical passages and did not acquire the mutations thus, we rule out the possibility of

long-term mechanical passages inducing the nonsynonymous mutations observed in DAGK, PANK1, and PI4Kβ.”

1. The authors need to include the number of technical/ biological replicates and statistical evaluation in figure legend 1.
2. The pictures of the gels in Figure 2 can be condensed to only show the respective bands, with the rest of the gel being cropped out.  The secondary maker lane can be removed.
3. Tables with PCR primer sequences and PCR amplification conditions can be moved to a supplementary file.
4. Mutations cannot be “toxic” to parasites, they can incur a cost to fitness phenotype or if the gene product is essential to the parasite, parasite growth will be refractory. “CK and CDPK1 are essential for the growth of the asexual blood-stage parasites; thus, the acquisition of a mutation at the functional motif may be toxic for parasites”. Furthermore, the authors also need to include a ref for the essentiality of CK and CDPK genes.
5. I think that the following conclusion is unjustified: “and thus likely mediators of drug responses”. This is based on the fact that the authors do not show any drug response effect with known kinase inhibitors. This statement should be verified with a reference at least.
6. My last major concern is the context of the qPCR data. I do not think that that the paper provides sufficient proof or biological context to validate that the mutations of the specific kinases would influence their expression profiles, and how this relates to AQ resistance.  Context can be provided if the expression profiles of genes conveying the AQ resistance was perhaps included, and this was provided in context with literature with some means of a biological explanation. The SD deviations provided by the qPCR is also more than >2/3 than the actual values, and the housekeeping gene data is not included.   The statistical determinants are not shown on the graph and the number of biological replicates is not indicated in the figure legend.

The manuscript: “Amodiaquine drug pressure selects nonsynonymous mutations in pantothenate kinase 1, diacylglycerol kinase, and phosphatidylinositol-4 kinase in Plasmodium berghei ANKA” evaluated the development of mutations to known ACT partner drugs in various key kinases in *P. berghei *ANKA parasites.

 From a biological perspective this link is interesting as the role of kinases and associated signaling cascades has not been investigated in P. falciparum drug resistant lines. There are some major points that I think the authors need to take into consideration, in order to substantiate their findings.   

 For the introduction section:

1. I would propose a rewrite of the second sentence in the introduction as follows: “Of the six species that infect humans, Plasmodium falciparum is the most virulent, **and is responsible for** majority of deaths.”
2. The sentence “LM is an aryalcohol drug, predicted to share mechanisms of action with 4-aminoquinoline9.” Can be moved up after the “Like chloroquine, AQ and PQ are aminoquinoline drugs predicted to exert their action within the parasite digestive vacuole by inhibiting toxic heme polymerization9.” sentence. 
3. The authors need to include examples or references to the following statement: “These studies also indicate that the mechanisms of AQ, PQ, and LM may involve other unexplored essential parasite proteins.”
4. I am unsure what the authors mean by the term “protein kinases are drug activators”? Perhaps include a reference to this.
5. Edit the following sentence as suggested: “In Plasmodium species, the protein kinases are drug activators, potential targets for **the** drug metabolites, and mediators of drug resistance22,32–34.”
6. I think the bolded number “37” may be a typographical error: “Other kinase based-inhibitors that have progressed to human clinical trials are phosphatidylinositol 4-kinase (PI4K), with inhibitor **37** (MMV048)23,37.”
7. The statement highlighted in point 8, above, can also include the following citation: “Brunschwig C, Lawrence N, Taylor D, Abay E, Njoroge M, Basarab GS, Le Manach C, Paquet T, Cabrera DG, Nchinda AT, de Kock C, Wiesner L, Denti P, Waterson D, Blasco B, Leroy D, Witty MJ, Donini C, Duffy J, Wittlin S, White KL, Charman SA, Jiménez-Díaz MB, Angulo-Barturen I, Herreros E, Gamo FJ, Rochford R, Mancama D, Coetzer TL, van der Watt ME, Reader J, Birkholtz LM, Marsh KC, Solapure SM, Burke JE, McPhail JA, Vanaerschot M, Fidock DA, Fish PV, Siegl P, Smith DA, Wirjanata G, Noviyanti R, Price RN, Marfurt J, Silue KD, Street LJ, Chibale K. UCT943, a Next-Generation Plasmodium falciparum PI4K Inhibitor Preclinical Candidate for the Treatment of Malaria. Antimicrob Agents Chemother. 2018 Aug 27;62(9):e00012-18. doi: 10.1128/AAC.00012-18. Erratum in: Antimicrob Agents Chemother. 2018 Oct 24;62(11): PMID: 29941635; PMCID: PMC6125526.”
8. Edit the following sentence as suggested: “ **Commonly,** G **g**enes and mutations mediating resistance to one class may be associated with cross-resistance to drugs of unrelated chemical classes and modes of action.”
9. Include a ref to the following sentence: “Commonly, genes and mutations mediating resistance to one class may be associated with cross-resistance to drugs of unrelated chemical classes and modes of action.”

Scientific results and methods

1. The observation that nonsynonymous mutations only occurred in AQr parasites is strange. This drug is of the same mechanism of action and chemical class as the PQ (8-aminoquinolines), yet no kinase specific mutations were observed in the PQr line. The authors need to provide some justification as to this observation. This is in line with the following sentence in the manuscript:” We thus propose that submitting AQ pressure on Plasmodium berghei ANKA is accompanied by the acquisition of mutation in PANK1, DAGK, and PI4Kβ.” The authors need to give some reference to any biological consideration that could justify this observation, and why specifically this occurs within a AQr line.
2. Furthermore, the authors showed no justification or validation that mutations are specific to kinases in the drug resistant AQr line. There is no sequencing data of other genes presented in this paper. I think that if the authors want to emphasize the synonymous mutation of the specific kinases they need to show WGS full genomic sequence analysis, for at least a couple of parasites. Or at least include several other genes in the PCR experiment. The authors even state this in the paper: “ The parasites likely possess mutations in other protein sections. Whole gene sequencing or whole-genome sequence of the parasites may reveal changes in other functional regions of the protein.”
3. The use of SI units and scientific nomenclature in the methods section is incorrect. Minutes should be min hour hour/hours are h, rpm’s are not SI units and need to be converted to x *g *(note italics of *g)*, ml and µl should be mL and µL, degrees should be °C, spaces should be included between all SI units.
4. All text the manuscript must be justified.
5. I think the authors needed to include some data as the viability of drug sensitive vs drug resistant parasites, either as a phenotypic evaluation or measurement of growth replication.
6. Most importantly to truly assess the effect of kinase specific mutations, drug shift assays with known kinase inhibitors with and without PQ, AQ and LM needed to be performed. This result is critical to this paper.
7. The following sentence is unnecessary:” At the same time, their respective resistant parasites had retained resistance phenotypes, suggesting that the resistant mechanisms were encoded within the parasite’s genome.” All mutations incurred in parasite drug resistant lines occur on a genomic level.
8. The following sentence needs to be rewritten: “The WT parasites passaged a similar number of mechanical passages and did not acquire the mutations thus, we rule out the possibility of

long-term mechanical passages inducing the nonsynonymous mutations observed in DAGK, PANK1, and PI4Kβ.”

1. The authors need to include the number of technical/ biological replicates and statistical evaluation in figure legend 1.
2. The pictures of the gels in Figure 2 can be condensed to only show the respective bands, with the rest of the gel being cropped out.  The secondary maker lane can be removed.
3. Tables with PCR primer sequences and PCR amplification conditions can be moved to a supplementary file.
4. Mutations cannot be “toxic” to parasites, they can incur a cost to fitness phenotype or if the gene product is essential to the parasite, parasite growth will be refractory. “CK and CDPK1 are essential for the growth of the asexual blood-stage parasites; thus, the acquisition of a mutation at the functional motif may be toxic for parasites”. Furthermore, the authors also need to include a ref for the essentiality of CK and CDPK genes.
5. I think that the following conclusion is unjustified: “and thus likely mediators of drug responses”. This is based on the fact that the authors do not show any drug response effect with known kinase inhibitors. This statement should be verified with a reference at least.
6. My last major concern is the context of the qPCR data. I do not think that that the paper provides sufficient proof or biological context to validate that the mutations of the specific kinases would influence their expression profiles, and how this relates to AQ resistance.  Context can be provided if the expression profiles of genes conveying the AQ resistance was perhaps included, and this was provided in context with literature with some means of a biological explanation. The SD deviations provided by the qPCR is also more than >2/3 than the actual values, and the housekeeping gene data is not included.   The statistical determinants are not shown on the graph and the number of biological replicates is not indicated in the figure legend.

Is the work clearly and accurately presented and does it cite the current literature?

Yes

If applicable, is the statistical analysis and its interpretation appropriate?

Yes

Are all the source data underlying the results available to ensure full reproducibility?

Yes

Is the study design appropriate and is the work technically sound?

Partly

Are the conclusions drawn adequately supported by the results?

No

Are sufficient details of methods and analysis provided to allow replication by others?

Yes

Reviewer Expertise:

Biochemistry, Parasitology, Molecular cell biology, in vitro cell culture, protein biochemistry, antimalarial drug discovery

I confirm that I have read this submission and believe that I have an appropriate level of expertise to state that I do not consider it to be of an acceptable scientific standard, for reasons outlined above.

# Reviewer report: Reviewer response for version 2

**DOI:** 10.21956/openresafrica.15087.r30208

**Published:** 2023-10-06

**Authors:** Din Syafruddin Eijkman

**Affiliations:** 1 University of Hasanuddin, Makassar, Indonesia

**General comment:**

 Using the *in silico* approach, the authors tried to identify the molecular basis for the amodiaquine resistance. Unfortunately, the authors claim that amodiaquine drug pressure selects for the nonsynonymous mutations in the aforementioned genes is baseless as the authors did not perform the required experiment to justify the claim. Therefore, findings on the nonsynonymous mutations and their association with amodiaquine resistance should be proven using either “reverse genetics or in vivo sublethal challenge of *P. berghei* ANKA with amodiaquine to raise amodiaquine-resistant strain.

**Specific comment:**

 The nonsynonymous mutations identified by the authors in amodiaquine-resistant P. berghei ANKA could be proposed as the underline molecular basis for amodiaquine resistance but further evidence is required to prove the phenotype-genotype association.

**Suggestion:**

 The authors may re-check their data on the amodiaquine-resistant *P. berghei* they have, to see the level of resistance and the presence of (or selection for) the nonsynonymous mutations in the aforementioned gene.

**Recommendation:**

 The manuscript is approvable but requires major revision.

Is the work clearly and accurately presented and does it cite the current literature?

Partly

If applicable, is the statistical analysis and its interpretation appropriate?

Not applicable

Are all the source data underlying the results available to ensure full reproducibility?

Yes

Is the study design appropriate and is the work technically sound?

Partly

Are the conclusions drawn adequately supported by the results?

No

Are sufficient details of methods and analysis provided to allow replication by others?

No

Reviewer Expertise:

NA

I confirm that I have read this submission and believe that I have an appropriate level of expertise to confirm that it is of an acceptable scientific standard, however I have significant reservations, as outlined above.

# Response

**Published:** 2023-10-14

**Authors:** Daniel Kiboi

**Affiliations:** Jomo Kenyatta University of Agriculture and Technology, Kenya

**General comment:** Using the *in silico* approach, the authors tried to identify the molecular basis for the amodiaquine resistance. Unfortunately, the authors claim that amodiaquine drug pressure selects for the nonsynonymous mutations in the aforementioned genes is baseless as the authors did not perform the required experiment to justify the claim. Therefore, findings on the nonsynonymous mutations and their association with amodiaquine resistance should be proven using either “reverse genetics or in vivo sublethal challenge of *P. berghei* ANKA with amodiaquine to raise amodiaquine-resistant strain. **Author’s response** The *in silico* approach was primarily employed at the beginning of the study for the sole purpose of predicting and identifying conserved motifs/regions such as ligand binding sites or active sites in the protein sequences of CK, PANK1, DAGK, PI4Kβ, and CDPK1. These motifs were prioritized during PCR amplification and sequencing in the study. The claim that amodiaquine drug pressure selects non-synonymous mutation was only drawn from the analysis of the PCR amplified and sequenced fragments of the target regions from the amodiaquine-resistant lines and compared with the drug-sensitive wild-type (WT) parasites. We agree with the reviewer that reverse genetics experiments are required to validate the role of the identified non-synonymous mutations in mediating amodiaquine resistance or susceptibility in *P. berghei*. In our conclusion, we have edited this section for clarification. These reverse genetics were beyond the scope of this study but are currently under consideration. Finally, the amodiaquine-resistant strain was previously selected after submitting *P. berghei* ANKA parasites in mice and treated with amodiaquine. This selection of resistant parasites was achieved after many mechanical passages and using different drug concentrations (Kiboi et al. 2009; Ndungu et al. 2018). Before using these parasites in this study, the resistant profile of amodiaquine-resistant strain against amodiaquine was reevaluated, confirmed, and is described in the results section, “Drug response profiles confirmed the stability of the LMr, AQr, and PQr parasite.” **Reviewer's Specific comment:** The nonsynonymous mutations identified by the authors in amodiaquine-resistant *P. berghei* ANKA could be proposed as the underline molecular basis for amodiaquine resistance, but further evidence is required to prove the phenotype-genotype association. **Author's response ** We agree with the reviewer and, as previously stated in the last section of the abstract, that the role of the mutations in the resistant parasites and their impact on drug responses will require further investigations in malaria parasites. We have slightly edited that statement of the abstract for better clarity and emphasized it in the conclusion section at the end of the manuscript. **Reviewer's Suggestion:**

 The authors may re-check their data on the amodiaquine-resistant *P. berghei* they have to see the level of resistance and the presence of (or selection for) the nonsynonymous mutations in the aforementioned gene. **Authors response:** As stated earlier in our first response comments, before using the resistant parasites for PCR amplification and sequencing to evaluate for polymorphisms in the targeted genes, the resistance profiles were assessed in parallel with the control (drug-sensitive wild-type parasites). This is illustrated in Figure 1 and described in detail in the results section on “Drug response profiles confirmed the stability of the LMr, AQr, and PQr parasite.” In summary, using different drug concentrations, the resistant parasites grew and multiplied in the presence of the drug, while the drug-sensitive wild-type parasites were suppressed. These data confirmed that resistant parasites had retained the resistance phenotype. During the selection of amodiaquine-resistant parasites (Kiboi et al 2009; Ndungu et al 2018) and as stated in the results, the drug-sensitive wild-type parasites were passaged in mice without the drug pressure for an equivalent number of mechanical passages and did not acquire these non-synonymous mutations; therefore, we ruled out the possibility of long-term mechanical passages inducing the nonsynonymous mutations observed in DAGK, PANK1, and PI4Kβ.

# Reviewer report: Reviewer response for version 1

**DOI:** 10.21956/openresafrica.14585.r29370

**Published:** 2022-12-01

**Authors:** Lai Ngit Shin

**Affiliations:** 1 Institute for Research in Molecular Medicine (INFORMM), Universiti Sains Malaysia, Gelugor, Malaysia

The manuscript is accepted for indexing with some minor modifications as follows:

 Abstract:

1. Why were CK and CDPK1 missing from mRNA measurement despite probe for SNPs?

Introduction:

1. Well written.
2. I suggest to include a figure to illustrate the link between protein kinases in mediating drug action and resistance.

Methods:

1. Typo error - "malaria gas"; change to "gas mixture".
2. Give the details of the equipment used in the experiment which is missing in the manuscript. E.g. QuantStudio™ 5 System qPCR machine.

Results & Discussion:

1. There are missing marker labels in Figure 2.
2. The authors should include a figure to explain the key findings from this research especially involved with PANK1, PI4Kβ, and DAGK.

Acknowledgment:

- Provide the source of funding and declaration of conflict of interest among all authors.

Is the work clearly and accurately presented and does it cite the current literature?

Yes

If applicable, is the statistical analysis and its interpretation appropriate?

Yes

Are all the source data underlying the results available to ensure full reproducibility?

Yes

Is the study design appropriate and is the work technically sound?

Yes

Are the conclusions drawn adequately supported by the results?

Yes

Are sufficient details of methods and analysis provided to allow replication by others?

Yes

Reviewer Expertise:

Molecular biology

I confirm that I have read this submission and believe that I have an appropriate level of expertise to confirm that it is of an acceptable scientific standard.
